# A proposed mobility and resource prediction method for seamless handover and service continuity in 5G small cell networks

**DOI:** 10.1371/journal.pone.0355372

**Published:** 2026-08-12

**Authors:** Khoa Nguyen Dang Dinh, Peppino Fazio, Miroslav Voznak

**Affiliations:** 1 Department of Telecommunication, VSB-Technical University of Ostrava, Ostrava, Czechia; 2 Department of Molecular Sciences and Nanosystems, Ca’ Foscari University of Venice, Venice, Italy; Dr Shakuntala Misra National Rehabilitation University, INDIA

## Abstract

Developments in 5G New Radio (NR) aim to provide the best possible user experience, but barred and resource-depleted cells in dense small cell networks can cause service interruptions and increase handover latency. To address these challenges, we propose a proactive mitigation framework that applies the unified Autoregressive Recurrent Neural Network (AR-RNN). This framework simultaneously predicts a user’s future cell trajectory and their resource requirements by learning from historical data. By integrating these predictive forecasts with standard network-monitoring alerts (e.g., from an Intrusion Detection System (IDS), a Network Management System (NMS), or Operations, Administration, and Maintenance (OAM) systems), the system preemptively reroutes users to avoid flagged or unavailable cells. Applying the algorithm to both Homogeneous and Heterogeneous networks, our simulations demonstrate that this proactive approach yields substantial and quantifiable improvements in network performance across both topologies. The AR-RNN model achieves a next-cell prediction accuracy of up to 95.8%, establishing high dependability. This accuracy directly translates to enhanced Quality of Service (QoS) by reducing handover latency even to as low as nearly 5 ms. Furthermore, the framework significantly improves network reliability, reducing the network outage probability by up to 50% compared to standard reactive handover procedures. These results demonstrate a concrete and effective method for creating a more seamless and efficient telecommunications experience in dense 5G environments.

## 1 Introduction

In the 5G network domain and beyond, the relationship between latency and throughput is a critical factor determining overall performance and user experience [1,2]. Latency, the data travel time, and throughput, the data volume transferred per unit time, are interdependent performance indicators. The need for substantial improvements in both has become increasingly pressing with the rise of contemporary real-time applications like virtual/augmented reality, autonomous vehicles, and remote surgery, which demand ultra-low latency and high throughput for instantaneous data exchange and control [3–5].

In this context, the deployment of small cells, including femtocells, which possess unique characteristics such as user-deployment, low power, and IP backhaul [6], is essential for evolving wireless communication networks, particularly 5G NR. These compact, low-power base stations (BS(s)) enhance network capacity and localized coverage, especially in areas with high user density, by offloading traffic from macrocells [3–5]. Their contribution enables network densification, supports higher data rates, improves Quality of Service (QoS), and facilitates core 5G use cases like enhanced mobile broadband and ultra-reliable low-latency communications.

Despite these benefits, small cells, especially femtocells, inherently possess drawbacks related to lower power and shorter coverage ranges compared to microcells or macrocells. These constraints limit the number of users served per cell and necessitate frequent handovers as UEs move through densely deployed environments. These challenges, inherent to dense femtocell deployments as surveyed in [6], become particularly problematic when the target cell is unexpectedly barred (due to maintenance, faults, or severe overload) or experiences sudden resource depletion. Such events can lead to abrupt service disruptions, failed handovers, or severe QoS degradation, undermining the critical low-latency and high-reliability goals of 5G, especially for demanding interactive applications. This challenge of maintaining service continuity for individual users represents a form of micro-resilience at the network edge. It is a complementary problem to the broader field of macro-resilience, which focuses on safeguarding large-scale network infrastructure against catastrophic events. Significant research in macro-resilience has explored the topological vulnerability of entire networks, such as Earth orbit satellite constellations under geographical failures [7] or the design of disaster-resilient terrestrial backbones with risk-zone-diversified paths [8]. These vital works focus on robust network planning and design to ensure the core infrastructure can survive widespread damage. In contrast, our research addresses the dynamic, real-time operational challenge of managing the high-frequency, localized unavailability of individual cells due to transient conditions like resource depletion or temporary barring. Our work, therefore, focuses on an intelligent, user-centric service continuity mechanism designed to operate within an already established network topology.

Several general approaches aim to mitigate small cell limitations. Traditional mobility management algorithms focus on optimizing handover decisions based on current measurements [9–11]. HetNet integration strategies combine small cells with macrocells for better coverage continuity [12]. Advanced antenna techniques like beamforming can improve small cell coverage and capacity [13]. A fourth category involves predictive mobility and resource mechanisms [14–17], which anticipates UE movement and requirements. This predictive approach is particularly promising as it can potentially enhance network management by integrating intelligence into the existing infrastructure without necessarily requiring major hardware or protocol modifications.

Therefore, enhancing the network’s ability to intelligently select a predictively viable target cell and prepare handover procedures in advance is essential for ensuring seamless transitions and maintaining consistent QoS in dynamic small-cell environments. Mobility prediction, predicting a user’s future location or trajectory, has been extensively researched [14,18]. Early techniques adapted for cellular networks include probability-based methods like Markov chains [17,19–23] and state-estimation approaches like Kalman filtering [24–28]. While these foundational methods are effective in macro-cell environments with predictable, wide-area movements, they exhibit significant drawbacks in the context of dense, femto-like small cell networks. Such as, traditional Markov models are limited by their “memoryless” property, where the next predicted cell depends only on the current cell. This fails to capture the complex, path-dependent nature of user trajectories. Furthermore, their reliance on static transition probabilities makes them unsuitable for the dynamic and context-dependent mobility patterns found in urban environments. The Kalman filters are excellent for linear systems but struggle with the highly non-linear nature of movement in dense urban areas (for example: sharp turns, abrupt stops, ...); More advanced Kalman filter methods, such as Extended Kalman filter, although able to deal with non-linear movement, require not only the non-linear physical model but also its Jacobian matrix, which abrupt maneuvers (for example: sharp turns), making it infeasible to deal with a large case of complex trajectory movements in practice. More fundamentally, they operate on continuous coordinates, where even minor prediction errors can lead to assigning the user to the wrong cell entirely — a critical issue that our proposed cell-ID-based approach is designed to mitigate.

The evolution of machine learning brought forth techniques like SVR [29,30], Decision Trees [31], and XGBoost [32], which were applied both to location prediction and related tasks like estimating user traffic requirements [31].

More recently, deep learning, particularly Recurrent Neural Networks (RNNs) like LSTMs and GRUs [33], has shown significant potential for modeling sequential mobility data. For example, Meneghello et al. [15] combined GRUs, CNNs, and Markov Chains in a hybrid model to predict the next serving base station with high accuracy (>88%) based on radio signal characteristics. Convolutional Neural Networks (CNNs) have also been explored, such as the work by Fazio et al. [16], which investigated the encoding of mobility trajectories as images for next-cell prediction. These advanced approaches [34–37] demonstrate the power of deep learning in handling complex mobility patterns.

However, despite these advancements in prediction accuracy, critical gaps remain concerning proactive handover management, specifically aimed at preventing disruptions in dense small cell networks. Firstly, the majority of existing mobility prediction methods rely on coordinate-based trajectory prediction [38–46]. As discussed in the Mobility problem subsection, this approach can suffer from error accumulation and drifting effect, significantly increase the prediction error in dense networks with small cells or femtocells. Such environments, whose unique deployment characteristics are discussed in [6], where inaccuracies can lead to predicting the wrong cell entirely. Secondly, a robust proactive handover requires anticipating not only the next cell but also the resource requirements the UE will need upon arrival. While traffic prediction exists [31], the seamless integration of resource prediction with cell-sequence prediction, specifically for handover preparation, is less explored. Thirdly, while prior works like [15,16] focus effectively on optimizing next-cell prediction accuracy, they do not propose or evaluate an integrated framework that explicitly uses these predictions to proactively identify and avoid anticipated cell barring or resource depletion, as well as prepare the resources at alternative viable cells.

To address these limitations, this paper proposes a cell-based mobility and resource prediction method that uses a unified autoregressive RNN (AR-RNN). By directly forecasting the sequence of future cell IDs a UE is likely to visit, our approach inherently mitigates the drift and error accumulation issues associated with coordinate-based methods in dense environments. Unlike the hybrid model in [15] or the image-based CNN approach in [16], we employ a single AR-RNN architecture (detailed in the Model and Scheme for Mobility Prediction and Resource Prediction section) to forecast both the future cell sequence (in Equation 3) and the corresponding resource block requirements (in Equation 5). This leverages the sequential modeling strengths of RNNs directly on cell transition and resource usage data. Furthermore, our work includes a comparative evaluation against multiple baseline techniques (Table 3 in the Comparison and selection of models for the proposed algorithm section) to validate the suitability of AR-RNN for these combined prediction tasks.

The primary goal and core contribution of this study lie in demonstrating how integrating this unified cell-based mobility and resource prediction into standard network-controlled handover and resource allocation procedures allows for a truly proactive management scheme. This scheme helps the network (as depicted in the flow chart in Fig 7) anticipate future cell conditions, actively identify cells predicted to experience barring or resource depletion, select the most suitable alternative target cell in advance, and prepare handover procedures and necessary resources before the UE encounters problems [47,48]. This proactive framework aims to minimize problematic handovers, reduce latency, improve throughput, and lower outage probability compared to traditional reactive approaches in dense 5G small cell/femtocell networks, thus significantly enhancing the user experience.

The proposed cell-based prediction method for 5G small cell networks offers several key benefits:

- Reduction in network latency by preparing anticipated network handovers before UE transitions between cells.
- Assistance to the network through multistep and high-accuracy predictions of viable cells. The network has backup cell options for the UE if the initially selected cells encounter problems.
- A solution for small cell networks. Compared to a Euclidean trajectory-based prediction method, a cell-based approach is especially effective in small cell networks in minimizing the effect of common flaws in time series and regression algorithms:
  - Error accumulation: Errors in any given step can propagate and amplify in subsequent steps.
  - Drifting effect: Over time, predictions can deviate from actual values, producing inaccuracies.

Although such errors have an insignificant impact on networks with large coverage cells, the effects compound in networks with short-range cells. In small-cell scenarios, even slight deviations from the actual value of a prediction can result in the UE being incorrectly positioned and placed in the wrong cell.

## 2 Context and definition of the problem

This section outlines the context in which the proposed predictive mobility and resource approach was tested and simulated.

### 2.1 Mobility problem

Mobility prediction involves the use of historical trajectory data and mobility patterns of objects to predict their future movements. Trajectory types vary depending on the specific aims and objectives of the approach to address the problem at hand, but the most widely used is the global positioning system (GPS) trajectory (i.e., Euclidean, geographical trajectory). This type of trajectory data effectively describes the movement patterns of objects and is straightforward to collect using GPS-enabled devices.

**Fig 1:**
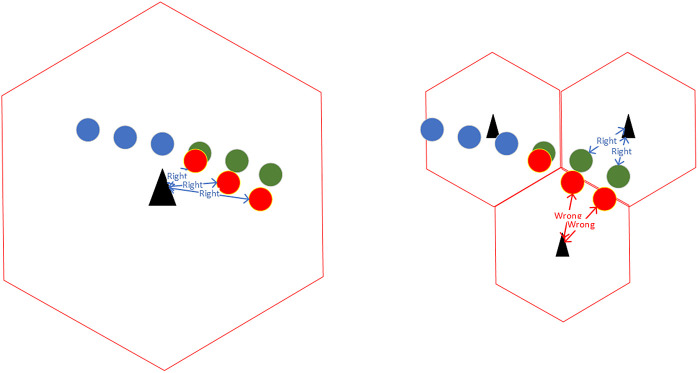
Illustration of the limitations of trajectory-based prediction compared to cell-based prediction.

In telecommunications, GPS trajectory data is widely applied in projects and studies that focus on solutions to a range of problems associated with predictive handover, predictive transmission conditions, and predictive user behavior. Although these applications have proved effective in large-cell environments such as macrocells and microcells, they encounter major limitations when applied in small-cell scenarios such as femtocells. The core issues arise from the inherent characteristics of regression and time-series models commonly used for prediction. These models are prone to well-documented problems, for example, the error accumulation, where errors at any given step can propagate and amplify in subsequent steps, and the drifting effect, where predictions can deviate from actual values over time. In large cells, the impact of these problems is minimal over a broad coverage area, but in small cells, the effects compound and can produce large inaccuracies. Even minor deviations in prediction can result in UE being incorrectly positioned, potentially distant, and in the wrong cell. This trajectory-based limitation is visually illustrated in Fig 1.

For small cell networks, we propose the use of cell-based trajectories, a novel type of trajectory data that incorporates the unique characteristics of these environments. Unlike Euclidean coordinate trajectory-based prediction methods, the cell-based approach is suited to small-cell scenarios, being more efficient in this type of environment and mitigating the errors associated with these effects.

### 2.2 Definition of cell-based mobility prediction

The problem is defined under the assumption that it reflects the real-life operations of contemporary cellular networks. The problem addressed in this work focuses specifically on improving connected-mode handover performance in dense cellular networks through proactive management, leveraging the cell-based trajectory approach proposed in the Mobility Problem subsection.

**Fig 2:**
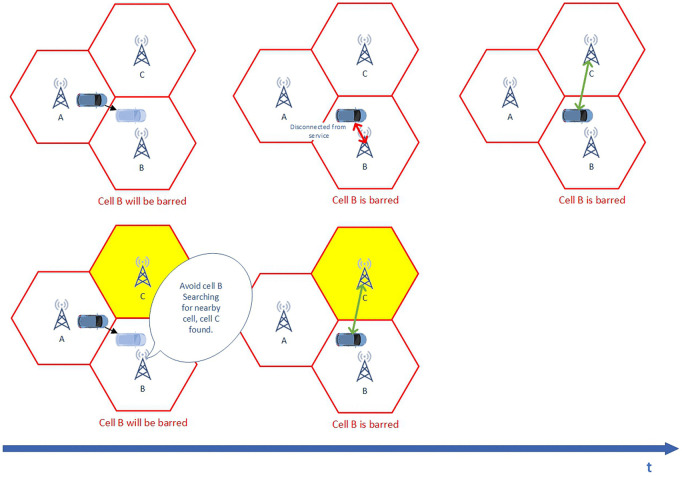
Illustration of the standard handover process in a cellular network when a barred cell is anticipated.

**Fig 3:**
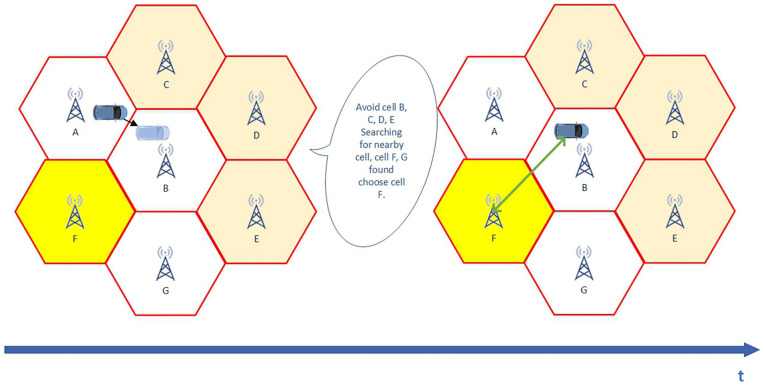
Illustration of the handover process incorporating the proposed mobility prediction method when a barred cell is anticipated.

We consider scenarios where potential handover target cells may become unsuitable due to various dynamic factors. These include imminent cell barring (which the network might predict based on operational data, scheduled maintenance, or escalating fault conditions, potentially before this status is reflected in the broadcast Master Information Block (MIB) cellBarred flag available to UEs) or anticipated severe resource depletion/congestion (predicted based on traffic analysis and the incoming UE’s forecasted resource demands using methods like the one proposed in the Definition of Resource Prediction section). While standard Radio Resource Management (RRM) reacts to current measurements and MIB status, our proposed method operates on the principle of proactive network resource management, aiming to predict these problematic conditions along a UE’s trajectory before a handover decision is finalized. When the network predicts, using the algorithms detailed in the Model and Scheme for Mobility Prediction and Resource Prediction section, that a potential target cell along the UE’s trajectory is likely to become unsuitable (due to predicted barring or resource depletion), it proactively scans for nearby, viable alternative cells. Based on this proactive evaluation, the network selects an optimal alternative target cell and initiates preparations, including requesting resource pre-allocation and/or reservation at the target cell and potentially initiating context transfer from the serving cell, before the UE needs to transition. This proactive preparation yields several key benefits. Firstly, the network can proactively avoid commanding the UE (in connected mode) to hand over to cells identified as potentially unsuitable (for example, those predicted to be barred or unable to meet the UE’s resource requirements upon arrival), thus preventing likely handover failures or immediate QoS degradation. Secondly, by preprocessing handover and resource allocation, the time required for the UE to establish a connection after transitioning is significantly reduced, enabling quicker, seamless connectivity, especially beneficial for UEs that transition frequently between cells. Figs 2 and 3 contrast this proactive approach with standard procedures.

Fig 2 highlights the contrast between the occurrence of barred or resource-depleted cells in a network using typical handover procedures and a network using mobility prediction to establish the connection. The process can be described as follows:

- The UE moves from its current cell, A, to cell B. Based on transmission reports and measurements, the network identifies (reactively based on current measurements) that cell B is likely to become barred, but standard procedures may not allow sufficient time for preventive action before the handover command to cell B is necessary or issued.
- Cell B becomes barred during the UE’s transmission.
- The UE is forced to select another nearby cell, in this case, cell C, resulting in a service interruption while handover and resource computations are performed at cell C. If cell C also becomes barred, the process repeats, with each iteration causing increased service interruptions.

By contrast, the procedure using the mobility prediction method is as follows:

- When the UE is in its current cell, A, the mobility prediction model forecasts the next cell to which the UE would likely connect, in this case, cell B. Simultaneously, based on transmission reports and measurements, the network identifies based on information of the system that cell B is likely to get problematic. The network proactively instructs an alternative suitable cell C to prepare for handover.
- When cell B becomes barred during the UE’s transmission, the network has already prepared the handover to cell C, enabling the UE to smoothly transition to cell C, bypassing the problematic cell B entirely and avoiding the time-consuming operations associated with handover failure and recovery.

The proposed model continuously scans for potentially problematic cells while the UE remains in its current cell. In the event of multiple potentially unsuitable cells, the network scans for the most suitable functional cell among all the in-range options. For example, as illustrated in Fig 3, if cells C, D, and E exhibit signs of potential cell barring, the network proactively identifies the best available functional cell, such as cell F. By the time the UE arrives at the initially barred or resource-depleted cell B, and other nearby cells such as C, D, and E are also compromised, cell F has already been selected and prepared. The UE will connect to cell F immediately, bypassing the time-consuming process of sequentially searching for and attempting to connect to multiple cells.

The cell-based mobility prediction method can be summarized into the following steps:

1. Convert Euclidean (or geographic) coordinates into cell-based coordinates to map the current UE position and associated cell.
2. Forecast the next set of cells, up to *b*, that the UE is likely to move towards.
3. Evaluate the predicted cells for potential compromise, such as cell barring or high traffic, and proactively select alternative cells if necessary.

*Definition 1:* The mobility prediction problem.

Given that each user is identified by its unique UE ID, let the m^*th*^ user be represented by their corresponding m^*th*^ ID, where the total number of users is M. We define $U_{m}=\left(u_{1},u_{2},...u_{m}\right)$ as the vector containing all user IDs $u_{1},u_{2},...u_{m}$, with $\left\|U_{m}\right\|=M$. It is assumed that $C_{m}^{\left(t\right)}$ is the vector containing the cell coordinates visited by the m^*th*^ user at time step *t*. The trajectory of the m^*th*^ user, based on the cells they are associated with, can be expressed as:


$$\begin{array}{c} {Trajectory}_{m}=\left(u_{m},C_{m}^{\left(t\right)}\right) \end{array}$$ (1)

Training the model on the entire trajectory dataset for each user requires significant memory and computational resources. To address this, the trajectory can be divided into smaller segments using the sliding window method. By selecting segment lengths that are sufficiently large to capture the trajectory’s overall pattern, the model can learn from the segmented data and predict subsequent locations with low error. We define the time range $\left[b_{1},b_{2}\right]$ as a sliding time window over [0, t], where b is the number of look-back steps, such that 0<b<t and b = b_2_-b_1_.

Let $C_{m}^{\left(b\right)}=\left(c_{m}^{\left(b_{1}\right)},...,c_{m}^{(b_{2})}\right)\;$ represent a subset of the cells visited by the m^*th*^ user along a route, derived from the corresponding segments of the user trajectory. The trajectory segment of the m^*th*^ user can then be defined as:


$$\begin{array}{c} {{Trajectory}_{m}}_{b}=\;(i_{m},\;C_{m}^{\left(b\right)}) \end{array}$$ (2)

The problem is then formulated as follows. Given a segment of *b* look-back cells that a user visited in the past, let *p* represent the probability of the user appearing at a specific cell, and let *a* denote the number of cells that the user is predicted to visit in the future. The user’s future visited cells is represented by $C_{m}^{\left(b_{2}+a\right)}=\left(c_{m}^{\left(b_{2}+1\right)},...,c_{m}^{(b_{2}+a)}\right)\;$, and the most likely sequence of future cells the user will visit is given by the equation:


$$\begin{array}{c} \hat{C_{m}^{(b_{2}+a)}}=\underset{{\;C}_{m}^{\left(b_{2}+a\right)}}{\underbrace{\mathrm{argmax}}}p\left(C_{m}^{\left(b_{2}+a\right)}\;|\;{{\mathrm{Trajectory}}_{m}}_{b}\right)\; \end{array}$$ (3)

### 2.3 Definition of resource prediction

In addition to mobility prediction, the study addresses the estimation of the radio resource blocks (RBs) a UE will require upon entering forecast future cells. This resource prediction task, when combined with the mobility prediction algorithm (in the Definition of Cell-based Mobility Prediction subsection), forms a comprehensive predictive framework, herein termed the Predictive Resource Management Algorithm. This framework aims to enhance traditional resource allocation, with particular relevance to proactive handover management in 5G femtocell networks.

The resource prediction problem is formulated similarly to the mobility prediction problem (Definition 1). Network operators can record historical RB demands of UEs, creating time-series data analogous to cell trajectories. Crucially, this allows the same type of deep learning methodology proposed for mobility prediction (detailed in the Model and Scheme for Mobility Prediction and Resource Prediction section) to be directly applied for predicting future resource block demands, simplifying both understanding and deployment.

*Definition 2:* The resource block prediction problem.

Let ${RB}_{m}^{\left(t\right)}$ denote the number of resource blocks (RBs) corresponding to the amount of bandwidth required by the $m^{th}\;$ user at the time *t*. Assuming a time interval Δt=1 second between each bandwidth measurement at the UE, we can define a discrete time series of the number of historical RB values as ${RB}_{m}^{\left(t-b\right)}=\left({rb}_{m}^{\left(b1\right)}...{bw}_{m}^{\left(b2\right)}\right)$, where $\left[b_{1},b_{2}\right]$ represents a sliding window within the interval [0, t], and b denotes the number of look-back steps, such that 0<b<t and b = b_2_-b_1_. Similarly, the future RB values, representing the predicted RB requirements, can be expressed as ${RB}_{m}^{\left(t+a\right)}=\left({rb}_{m}^{\left(b2+1\right)}...{rb}_{m}^{\left(b2+a\right)}\right)$. These values correspond to the predicted cells for the user, defined as $C_{m}^{\left(b_{2}+a\right)}=\left(c_{m}^{\left(b_{2}+1\right)},...,c_{m}^{(b_{2}+a)}\right)$. The predicted number of RBs required for the $m^{th}\;$ user with ID $i_{m}$ and associated with the predicted cells can then be described by the following formulation:


$$\begin{array}{c} {{Resource\;blocks}_{m}}_{b}=\;(i_{m},\;RB_{m}^{\left(b\right)}) \end{array}$$ (4)

$$\begin{array}{c} \hat{{RB}_{m}^{(b_{2}+a)}}=\underset{{\;RB}_{m}^{\left(b_{2}+a\right)}}{argmax\;}p\left({\;RB}_{m}^{\left(b_{2}+a\right)}|\;{{Resource\;blocks}_{m}}_{b}\right) \end{array}$$ (5)

This similarity simplifies both the understanding and the implementation of the proposed approach, highlighting its advantage as a solution.

## 3 Model and scheme for mobility prediction and resource prediction

To address the challenges outlined previously, this study proposes a predictive framework, termed the Predictive Resource Management Algorithm, designed for proactive handover and resource allocation in dense 5G small cell networks. This framework leverages predictions of both UE mobility and resource requirements to anticipate network conditions and guide handover decisions. The core tasks involve forecasting the sequence of future cells a UE is likely to visit (as defined in Eq. 3) and estimating the corresponding resource blocks (RBs) required in those cells (as defined in Eq. 5). Both prediction tasks are performed using a unified deep learning model based on an Autoregressive Recurrent Neural Network (AR-RNN), whose architecture is detailed below. The overall workflow, illustrated in Fig 7, involves using these predictions to evaluate potential target cells, avoid those predicted to be barred or resource-depleted, select a suitable alternative if necessary, and prepare the selected cell for the incoming handover.

**Fig 4:**
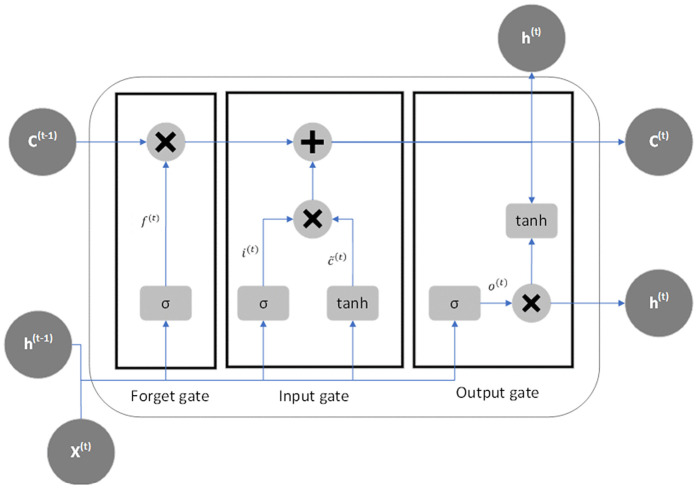
Structure of an LSTM cell.

The following paragraphs detail the AR-RNN model used for these predictions. As described in the Introduction, RNNs are powerful algorithms for sequential and time-series problems. The present study employs an autoregressive RNN (AR-RNN), an advanced variant of RNNs, to perform mobility and resource block prediction in an experimental simulation. The overall structure of the AR-RNN used in this work is depicted in Fig 4.

The AR–RNN incorporates several key components, outlined below:

1. **Input Layer**: The input layer is the network’s initial layer, responsible for receiving the input data, which could be a sequence of values or a single value in the case of autoregressive models.
2. **RNN layer (or LSTM/GRU Layer)**: This layer is the core of the network and is responsible for handling mathematical operations to examine sequential dependencies in the data. It comprises memory cells (typically LSTM or GRU cells).
3. **Dense Layer**: Like other neural network architectures, the AR–RNN has dense layers comprising fully connected neurons. These layers perform linear transformation followed by non-linear activation functions to generate meaningful representations and predictions in the final output.
4. **Output Layer**: The output layer generates the final predictions. The choice of activation function depends on the nature of the task. For regression problems, such as mobility and resource prediction, a linear activation function is employed. For classification tasks, a softmax activation function is used.
5. **Autoregressive Connection**: A feature of the AR-RNN is the autoregressive connection between the output and input layers. This feedback loop allows the model to use its own previous predictions as input for subsequent steps, enabling it to capture the dependencies between past and future values.
6. **Loss Function**: The loss function quantifies the error between the model’s predictions and the true values. It is used to optimize the network weights during training to minimize loss.
7. **Optimization Algorithm**: Optimization algorithms, such as gradient descent and its variants, are used to adjust the network weights iteratively. These adjustments are guided by the gradients calculated from the loss function.

These predictions are then used by the network to proactively select suitable handover targets, following the general workflow illustrated in Fig 7.

**Fig 5:**
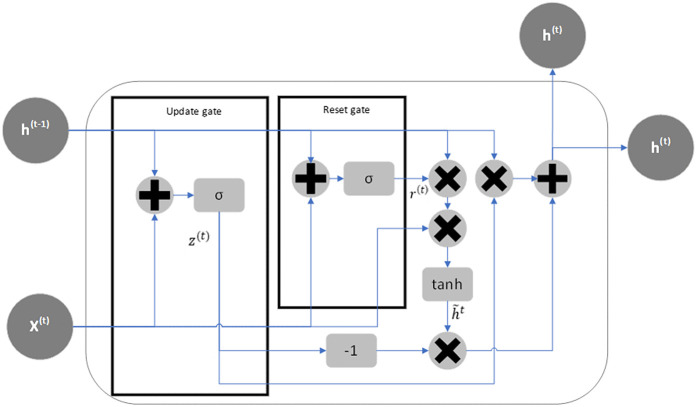
Structure of a GRU cell.

The RNN layer utilizes memory cells capable of capturing sequential dependencies. We explored both standard Long Short-Term Memory (LSTM) [49] (structure shown in Fig 4) and Gated Recurrent Unit (GRU) [50] (structure shown in Fig 5), as both are effective for time-series tasks. Both architectures use gating mechanisms to control information flow and learn long-range dependencies, updated via Backpropagation Through Time (BPTT). The choice between them often depends on the specific dataset.

According to the Figs 4, 5, we use the *x* as the input data, *h* as the hidden state, *c* as the cell state, *t* as the current time step, t−1 as the previous time step, σ as the sigmoid activation function, and *tanh* as the hyperbolic tangent activation function. For LSTM cell, we indicate new information to be written to the cell as ${\tilde{c}}^{\left(t\right)}$, the cell’s Forget gate as $f^{\left(t\right)}$, Input gate as $i^{\left(t\right)}$, and Output gate as $o^{\left(t\right)}$ For GRU, we denote the update gate and reset gate as *z*^(*t*)^ and $r^{\left(t\right)}$, and the candidate output of the current GRU cell as ${\tilde{h}}^{t}$.

LSTM and GRU owe their effectiveness to their ability to selectively update and forget information. This capability allows them to capture long-term dependencies in sequential data. The choice between LSTM and GRU depends on the specific task and dataset, as both have demonstrated effective performance for different scenarios.

**Fig 6:**
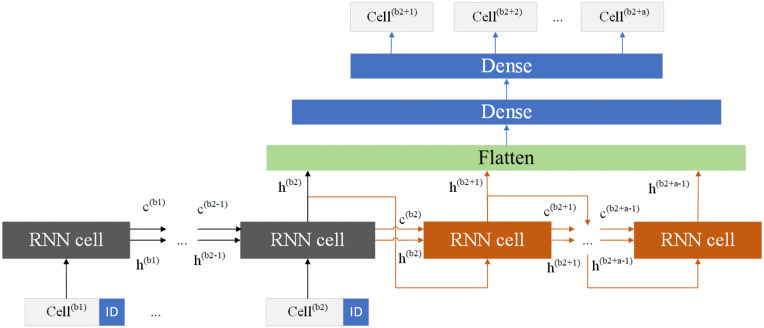
Structure of the autoregressive RNN.

Fig 6) illustrates the structure of the proposed AR-RNN. At the end of the RNN where input sequences are received, the autoregressive (AR) method (indicated as orange RNN cells) is applied to predict multiple subsequent states based on historical states.

Since the user ID and cell ID are independent variables, binary encoding is used to transform these identifiers into binary values of 0 and 1 [51–53] before incorporation into the model. Two notable advantages of this encoding method are: *One*, it eliminates the need for scaling to prevent gradient explosion [54], and *Two*, compared to the use of One-hot encoding (Such as in [15]), the binary encoding can reduce significantly the memory consumption, and allow to encode a larger number of BSs’ IDs without memory overflowing. These advantages contribute to allowing the used algorithm to handle a broader range of network cases, for example, 496634 BSs for the minimum 10m coverage radius, and 19887 BSs for the maximum 50m coverage radius in the Singapore city area of about 5500×2000 square meter scenario, compared to just 196 BSs with the cell’s coverage radius of 40m, in the Cologne city area of 900×700 meters square.

The model input comprises two branches. The first branch processes the categorical features, specifically the binary-encoded user IDs derived from the ID matrix. The second branch processes the binary-encoded cell ID features, denoted ${Cell}_{m}^{\left(b\right)}$, corresponding to the user. These two branches merge at the input to the RNN layers, where the encoded categorical and numerical feature vectors are combined into a matrix before being fed into the RNN. The output layer comprises *b* dense branches, corresponding to the *b* steps ahead that the model will predict. Each branch contains a number of neurons equal to the length of the vector of the cell ID features, ${Cell}_{m}^{\left(b\right)}$. The binary-encoded location, ${Cell}_{m}^{\left(b2+a\right)}$, is subsequently converted back to its original cell ID.

The Mean Square Error (MSE) is employed as the loss function to quantify the error between the actual values and the predicted values at the end of each training epoch. Assuming the dataset contains *M* samples, the MSE is calculated as follows:


$$\begin{array}{c} MSE=\;\frac{1}{M}\sum_{m=1}^{M}{(C_{m}^{(b_{2}+a)}\;-\;\hat{C_{m}^{(b_{2}+a)}})}^{2} \end{array}$$ (6)

This loss value is required for calculating the gradient used during backpropagation. After constructing the model architecture, various parameters are tested to identify the optimal configuration for the complete model. The MSE is again used to evaluate the performance of each model by measuring the mean squared difference between the predicted values, $\;\hat{Y_{m}^{(b_{2}+a)}}$, and the actual values, $Y_{m}^{(b_{2}+a)}$, across all users, expressed as:


$$\begin{array}{c} MSE=\;\frac{1}{M}\sum_{m=1}^{M}{(Y_{m}^{(b_{2}+a)}\;-\;\hat{Y_{m}^{(b_{2}+a)}})}^{2} \end{array}$$ (7)

To evaluate the performance of the model, the algorithm applies the *R*^2^ score metric. The *R*^2^ score ranges from 0 to 1, indicating how well the model’s predictions align with the actual values. It is expressed by:


$$\begin{array}{c} R^{2}=1-\frac{MSE(model)}{MSE(baseline)}=1-\frac{\sum_{n=1}^{N}{\left(Y_{n}^{\left(b_{2}+s\right)}-\hat{Y_{n}^{\left(b_{2}+s\right)}}\right)}^{2}}{\sum_{n=1}^{N}{\left(Y_{n}^{\left(b_{2}+s\right)}-Y^{\left(\bar{b_{2}}+s\right)}\right)}^{2}}, \end{array}$$ (8)

where $Y^{\left(\bar{b_{2}}+s\right)}$ is the mean of all $Y_{n}^{\left(b_{2}+s\right)}$. The closer the *R*^2^ score is to 1, the better the model performs in accurately predicting actual values.

**Fig 7:**
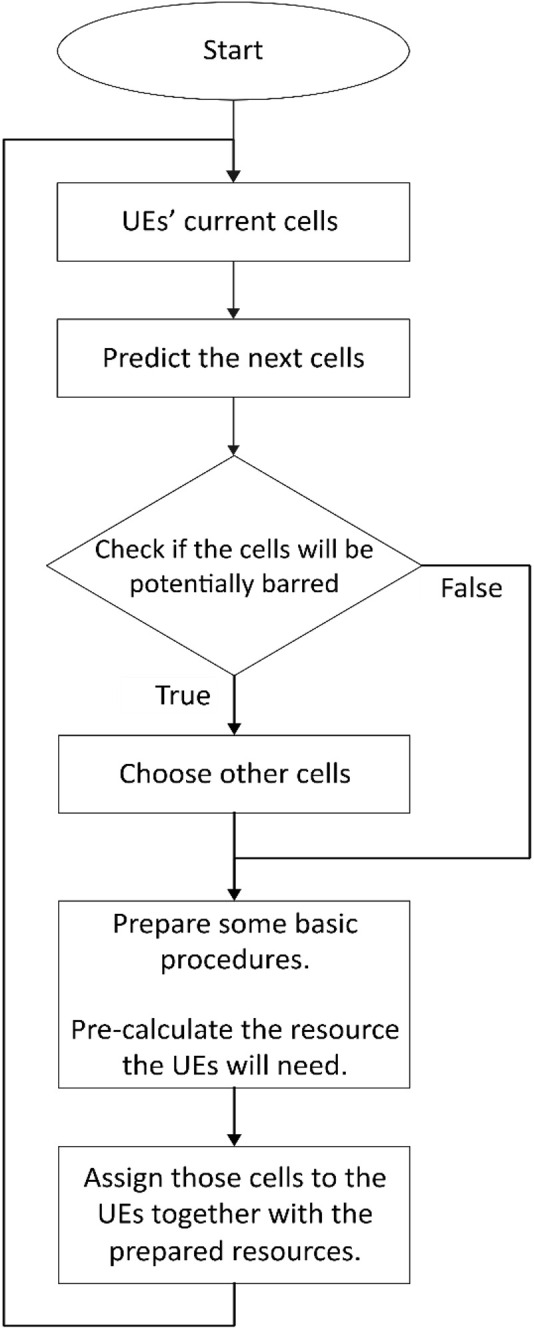
Workflow of the cell-based mobility and resource prediction algorithm.

The entire proposed resource management process based on mobility prediction is illustrated in the flow chart in Fig 7. As indicated by the chart, the scheme is designed to handle multiple users, processing each user individually until all users are completed. For each user, the network records the IDs of the cells they have visited, from t−b up to the current cell’s ID, t = 0. This data is then used to predict the next *s* cell IDs that the user is likely to visit in the upcoming *s* moments. The network then evaluates parameters such as the signal-to-interference-and-noise ratio (SINR), path loss, distance of the predicted cell, and the *x* number of nearby cells. The system selects the cell with optimal parameters to serve as the user’s incoming associated cell. This selected cell prepares the connection and allocates the bandwidth required by the user, as determined from Eq 5.

## 4 Simulation settings

As discussed in the Context and Definition of the Problem section, the study employs a cell-based mobility prediction algorithm to forecast the next cell that the UE would likely transition to in the near future. The proposed method operates under the premise that the network continuously monitors transmission reports and associated parameters to assess whether the predicted cell will likely experience cell barring. If the predicted cell is at risk of barring, the model assists the network in selecting a more suitable cell within the UE’s range at that moment. The selected cell is then prepared in advance for handover. When the UE reaches the barred cell, it automatically transitions to the new cell, connecting instantly with sufficient resources and bypassing the need for any further connection establishment procedures. Fig 7 illustrates the flow chart of the proposed method’s process, and also, for more clarification, we provide the pseudocode Algorithm 1 that shows how the process works.

**Algorithm 1 The proposed Predictive Resource Management Method**

**Require:** Current UE trajectory segment $Trajectory_{mb}=(i_{m},C_{m}^{\left(b\right)})$

**Require:** Network Status $N_{status}$ (barring info, available RBs for cells)

**Require:** Prediction Model $M_{AR-RNN}$

**Require:** Number of future steps to predict *a*

**Ensure:** Selected Target Cell ID $cell_{target}$, Estimated Required RBs $RB_{req}$

1: // Predict future cell sequence and required RBs

2: $C_{m}^{\left(b_{2}+a\right)}\leftarrow M_{AR-RNN}.predict\_mobility(Trajectory_{mb},a)$ ▷ Using Eq. 3 logic

3: $RB_{m}^{\left(b_{2}+a\right)}\leftarrow M_{AR-RNN}.predict\_resources(Trajectory_{mb},a)$ ▷ Using Eq. 5 logic

4: target_found←false

5: **for** s←1 to *a* **do** ▷ Iterate through predicted future steps

6:   $predicted\_cell\leftarrow C_{m}^{\left(b_{2}+a\right)}$ ▷ Get cell predicted for step *a*

7:   list_of_nearby_cell_ordered ▷ Get the collection of cells’ IDs nearby and including the predicted cell for step *a* in order from closest to farthest

8:   $predicted\_RBs\leftarrow RB_{m}^{\left(b_{2}+a\right)}$ ▷ Get RBs predicted for step *a*

9:   $is\_barred\leftarrow CheckBarringStatus(predicted\_cell,N_{status})$

10:   $is\_depleted\leftarrow CheckResource(predicted\_cell,predicted\_RBs,N_{status})$

11:   **for** cell∈list_of_nearby_cell_ordered **do**

12:     **if** NOT is_barred AND NOT is_depleted **then**

13:       $cell_{target}\leftarrow predicted\_cell$

14:       $RB_{req}\leftarrow predicted\_RBs$

15:       target_found←true

16:       **break** ▷ Found suitable predicted cell

17:     **else**

18:       $save\_RB_{req}\_for\_providing\_to\_the\_next\_cell$

19:     **end if**

20:   **end for**

21: **end for**

22: **if** NOT target_found **then** ▷ All predicted cells problematic

23:   $cell_{target},RB_{req}\leftarrow FindBestAlternativeCell(i_{m},N_{status})$ ▷ Scan nearby viable cells

24: **end if**

25: // Prepare handover to the selected target cell

26: $PrepareHandoverProcedures(cell_{target},i_{m},RB_{req})$

27: **return** $cell_{target},RB_{req}$

**Fig 8:**
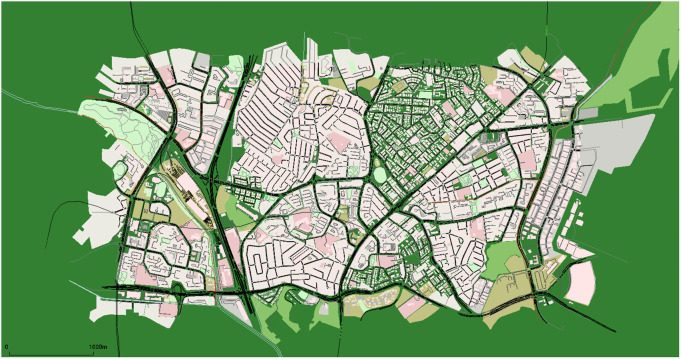
Simulation scenario generated from a real-world map of an area in Singapore.

**Fig 9:**
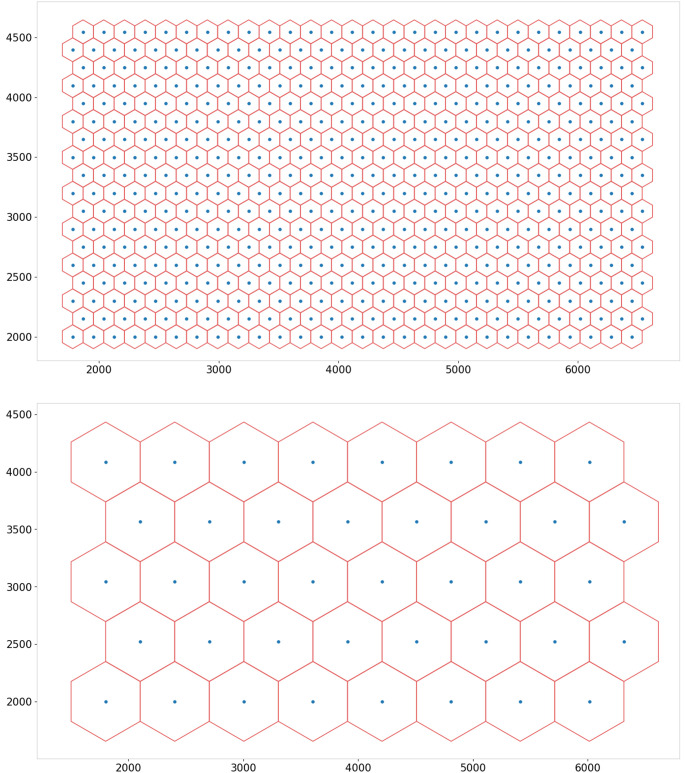
Layout of the simulated cellular network, with cells of different coverage radii.

Using SUMO and OSM wizard, we generated a simulation scenario from a real-world map of an area in Singapore, as shown in Fig 8. SUMO was then used to simulate vehicle movements within the scenario, representing mobile users in a practical setting, and to record their travel history as coordinate trajectories. After obtaining all user trajectory records, a network layout was created using Python and applied to the simulation to transform the position-based trajectories into their associated cell-based trajectories. To simplify the simulation, we specified an ultra-dense homogeneous network, where all cells are identical in type, range, and power. The network comprised femtocells capable of five different ranges, varying from 10 to 50 meters. For the purpose of the simulation, all cells were assumed to have uniform properties and technical parameters. The cell areas were modeled as ideal hexagons positioned adjacent for maximum coverage. Fig 9 illustrates the layout of the cellular network used in the simulation. This idealized homogeneous layout was intentionally chosen for these initial simulations to establish a controlled environment. It allows us to clearly evaluate the performance of the proposed cell-based prediction algorithms (AR-RNN) and the proactive management framework without the confounding factors of inter-layer interference and complex propagation variations inherent in more realistic heterogeneous network deployments. While we acknowledge that realistic femtocell deployments typically exhibit more irregular patterns and overlay structures, often utilizing user-provided backhaul, as comprehensively surveyed in [6], analyzing performance in a full HetNet scenario remains a key direction for our future work.

**Fig 10:**
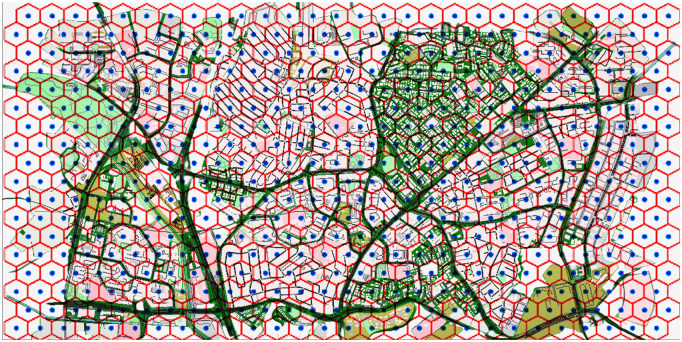
Illustration of a homogeneous femtocell network integrated into the simulation scenario.

Table 1 summarizes the parameter settings for several hundred users tracked in the simulation. To optimize simulation time and data collection, only 30 users were targeted as main subjects; the remaining users were generated and acted as random obstacles (Such as temporarily blocking the main users’ movement, or forcing the main user to slow down their speed,...) to simulate a real-world urban environment and also represent other random users trying to access the network. The experiment used three data sets for training, testing, and validation. These data sets were designed to simulate user travel on three different days along specific routes from start to destination. Table 2 outlines the transmission environment parameters used in the simulation. An illustration of this homogeneous femtocell network integrated into our simulation scenario is presented in Fig 10.

**Table 1:** Simulation parameter settings.

Simulation Parameters	Values
Area	An urban area in Singapore
Number of main considered vehicles	30 vehicles
GPS sampling time	1 sample/second
Average speed	≈30 km/h

**Table 2:** 5G NR system parameters.

Parameters	Values
Transmission environment	Homogeneous femtocell network, HgNB
Simulation scenario	Urban area taken from a map of Singapore
Channel Bandwidth	50 MHz
Carrier Bandwidth	2.6 GHz
Carrier Frequency	2 GHz
Subcarrier spacing	15 kHz
Guard band	692.5 kHz
OFDM Frame	10 ms
Slots	0.5 ms
Modulation Scheme	64 – QAM
Femtocell transmit power	20 dBm
Femtocell gain	5 dBi
Tx height	1 m
Rx height	1 m
Thermal noise	−174 dBm / Hz
RBs Bandwidth	180 kHz
Number of RBs	~ 270 blocks

## 5 Experimental results

### 5.1 Comparison and selection of models for the proposed algorithm

This section presents a comparison of the proposed model and other algorithmic approaches, including machine learning methods such as linear regression, decision trees, XGBoost, and probability-based methods such as the Markov chain model and Kalman filter.

Table 3 indicates that the probability-based models struggled to effectively handle the problem of multi-cell prediction for multiple users and exhibited limitations compared to the deep learning (DL) models. The proposed model AR-RNN with LSTM or GRU is shown to be the most effective of the DL models evaluated for this specific task. Both probability-based models demonstrated low accuracy (below 50%) and high numbers of prediction errors. The high *R*^2^ of some models is most likely because, despite the fact that they predict the wrong cell IDs, these wrong encoded IDs prediction approximate the true encoded IDs in terms of large values, so as a result, they show an unreliable high *R*^2^ while very high prediction error. By contrast, most of the DL models achieved accuracies exceeding 70%, some reach 90% and reasonably high *R*^2^ values. The simple CNN1D model shows an overall poor efficiency in all the cases of steps ahead, and gives the lowest prediction accuracy of 57.4%, the lowest *R*^2^ at 0.82, and the highest MSE of 0.04 among the DL models at 4 steps ahead. The chosen AR-LSTM model achieved overall good results from 1 to 4 steps ahead prediction, and get the highest accuracy of 95.8%, *R*^2^ is 0.99 and below 0.01 MSE at 1 step ahead. The DL models in overall, demonstrated their advantage over probability-based models, machine learning methods, and other approaches in handling multiple UE and multi-step prediction scenarios.

**Table 3:** Mobility models’ performance comparison across different steps.

	Steps											
Models	1			2			3			4		
	Acc	R²	MSE	Acc	R²	MSE	Acc	R²	MSE	Acc	R²	MSE
**Deep learning**												
AR – LSTM	95.8	0.99	0.00	94.3	0.98	0.00	89.1	0.96	0.01	81.6	0.94	0.015
AR – GRU	82.8	0.95	0.01	92.4	0.98	0.01	86.4	0.96	0.01	87.8	0.96	0.01
Conv1D - LSTM	78.8	0.95	0.01	88.4	0.97	0.01	81.6	0.98	0.01	80.6	0.94	0.02
CNN1D	70.9	0.87	0.03	76.4	0.93	0.02	66.1	0.74	0.07	57.4	0.82	0.04
DNN	83.3	0.95	0.01	84.9	0.96	0.01	68.4	0.72	0.06	82.1	0.94	0.02
**Machine learning**												
Linear regression	5e-3	0.99	4752e3		x			x			x	
Decision tree	6e-3	0.99	4753e3		x			x			x	
XGBoost	0	1.00	2555e3		x			x			x	
**Probability based**												
ARIMA	0.072	0.99	1478e6		x			x			x	
Markov model	0	0.00	1656e3		x			x			x	
Kalman filter	36e-5	1.00	2030e3		x			x			x	
CPT	0	0.98	1788e4		x			x			x	

A detailed examination of the results reveals that the LSTM and GRU RNN-based models achieved the lowest MSE, highest accuracies, comparable to the nonrecursive DNN and CNN1D models. The results indicate RNN-based models as the most suitable solution for sequential prediction problems, in this case, mobility prediction. The ability of RNN-based models to retain information from previous time steps and exploit that information in decision-making provided a large advantage in this context. By contrast, although the DNN and CNN1D models exhibited relatively high prediction accuracy, they were unable to retain past information and produced more prediction errors as the input sequence length increased.

For the resource prediction task, we proceed to use the same methods as the mobility for resource block prediction, with the only difference being employing the one-hot encoding for the application the users use, since the number of applications is much less than the number of cells, and it is suitable for a classification problem. We culminate in a comparison result as shown in the Table 4.

**Table 4:** Resource models’ performance comparison across different steps.

	Steps											
Models	1			2			3			4		
	Acc	R²	MSE	Acc	R²	MSE	Acc	R²	MSE	Acc	R²	MSE
**Deep learning**												
AR – LSTM	95.27	0.9	0.01	96.81	0.925	0.01	94.55	0.91	0.01	95.83	0.92	0.01
AR – GRU	94.44	0.89	0.01	95.75	0.91	0.01	93.67	0.87	0.017	91.20	0.83	0.0225
Conv1D - LSTM	93.89	0.87	0.02	40.30	0.76	0.03	74.56	0.457	0.067	94.17	0.88	0.013
CNN1D	66.11	0.28	0.09	47.88	0.93	0.01	84.00	0.67	0.03	96.76	0.93	0.01
DNN	89.17	0.74	0.03	40.30	0.69	0.04	85.22	0.643	0.04	68.15	0.34	0.078
**Machine learning**												
Linear regression	56e-4	0	4.52		x			x			x	
Decision tree	56e-4	0	4.52		x			x			x	
XGBoost	7.95	0.72	0.30		x			x			x	
**Probability based**												
ARIMA	89.37	0.82	0.194		x			x			x	
Markov model	79.28	0.041	1.763		x			x			x	
CPT	17.86	0	8.49		x			x			x	

Table 4 presents a comprehensive performance comparison of various models for the task of multi-step resource block (RB) prediction, a critical component of our proposed framework. The results provide a clear justification for the selection of an Autoregressive Recurrent Neural Network (AR-RNN) architecture, mirroring the findings from the mobility prediction task in Table 3.

A clear initial observation is the significant limitation of traditional Machine Learning and Probability-based models for this problem. As indicated by the ’x’ marks, these models are inherently designed for single-step forecasting and are not suited for the multi-step prediction required by our proactive system. Furthermore, their single-step performance is largely inadequate. For instance, Linear Regression and Decision Tree models show near-zero accuracy and R^2^ scores, indicating a complete failure to capture the data’s patterns. While XGBoost and ARIMA offer reasonable one-step performance, their inability to forecast further ahead makes them unsuitable.

In contrast, the Deep Learning models demonstrate a strong capability for multi-step forecasting. Among these, the proposed AR-LSTM and AR-GRU models emerge as the most effective and reliable choices. The AR-LSTM model delivers outstanding and remarkably stable performance, maintaining an accuracy of over 94% and an R^2^ score of 0.9 or higher across all four prediction steps, with a consistently low MSE of 0.01. Similarly, the AR-GRU model shows strong, consistent performance, with accuracy remaining above 91% and R^2^ above 0.83.

While other deep learning models show some potential, they lack the consistency of the AR-RNNs. For instance, the Conv1D-LSTM and DNN models exhibit a significant drop in accuracy at the 2-step prediction mark before recovering, indicating instability in their learning. The simple DNN model, lacking a recurrent structure to remember past states, shows a clear degradation in R^2^ as the prediction horizon extends, falling from 0.74 to 0.34.

In summary, the results for resource prediction in Table 4 strongly align with the findings for mobility prediction in Table 3. They confirm that the AR-RNN architecture (specifically with LSTM or GRU cells) is the superior choice for our unified framework, demonstrating the high accuracy and, critically, the stable multi-step forecasting performance required for a proactive handover management system.

This comparison was conducted using a 10-meter coverage radius. Given that the proposed method depends on cell-ID-based prediction, the coverage radii of individual cells had minimal to no effect on the prediction results. The relative performance differences in these approaches are consequently consistent for other coverage radii. Further testing with different coverage radii to determine the most effective model could therefore be skipped.

The experiment also shows the trade-off that exists between the computational expense of a prediction model and its accuracy benefits, particularly when comparing deep learning methods against traditional machine learning and statistical approaches:

Traditional Methods (Markov, Kalman, CPT and ML) Low Cost, Low Accuracy: The probability-based methods (such as Markov chains) and classical ML models (such as Decision Trees) evaluated in our study are computationally lightweight. Their training phases are typically fast, involving the calculation of transition probabilities or the fitting of models with relatively few parameters. For example, a Markov model’s primary “training” is simply counting transitions, and a Kalman filter’s update step is a series of efficient matrix multiplications. However, as demonstrated in our results, this computational efficiency comes at a steep price: their predictive performance is extremely poor, with accuracy for multi-step prediction often at or near zero. Their simplistic underlying assumptions (for example, the memoryless property of Markov chains or the linear system assumption of Kalman filters) prevent them from capturing the complex, non-linear dynamics of real-world user mobility.

Deep Learning (AR-LSTM, AR-GRU, DNN and CNN1D) High Investment, High Return: The proposed AR-LSTM model requires a significantly higher upfront computational investment. The training process is far more intensive due to two main factors: (1) a vastly larger number of trainable parameters within the LSTM cells, and (2) the iterative optimization process, which relies on computationally demanding algorithms like Backpropagation Through Time (BPTT) to learn long-range dependencies.

This higher computational expense, however, yields a crucial and dramatic return on investment. The AR-LSTM delivers a multi-step prediction accuracy that is orders of magnitude higher than the traditional methods, reaching up to 95.8% for one-step-ahead predictions. This superior accuracy is achieved precisely because the model’s complex architecture is designed to learn the very temporal, non-linear patterns that simpler models cannot. For the problem of proactive handover in dense 5G networks, where a single prediction error can lead to a service interruption and severe QoS degradation, this trade-off is clear and justifiable. It is critical to distinguish between the offline training cost and the real-time inference cost. While the training is heavy, the inference cost of the trained AR-LSTM model is a series of fast, parallelizable matrix operations. This marginal real-time computational expense is a small price to pay for the immense gain in prediction accuracy. The substantial improvements in network reliability (reduced outage) and QoS (lower latency) overwhelmingly justify the higher offline computational investment required by the deep learning approach.

In summary, the DL models exhibited superior performance in solving the complex task of cellular mobility prediction for multiple users. Among the evaluated approaches, the proposed AR-LSTM models were shown to be the most effective. The advantages of the DL models in general, and specifically the proposed model, enabled the network systems to fully exploit the UE mobility information stored in a database. The network was able to accurately predict the future cells to which the UE would likely connect, prepare for handover, and calculate the UE’s required resources, with minimal error. The time required for communication between a standard cellular network and UE was consequently reduced, raising the QoE for users.

### 5.2 Parameter analysis

The subsections here present experimental results and examine the proposed model’s training parameters in relation to its prediction performance with different look-back steps and cell radii. Throughput, latency, and the practical results of different vehicles are also analyzed. Finally, the proposed model is compared with other regression algorithms.

**Fig 11:**
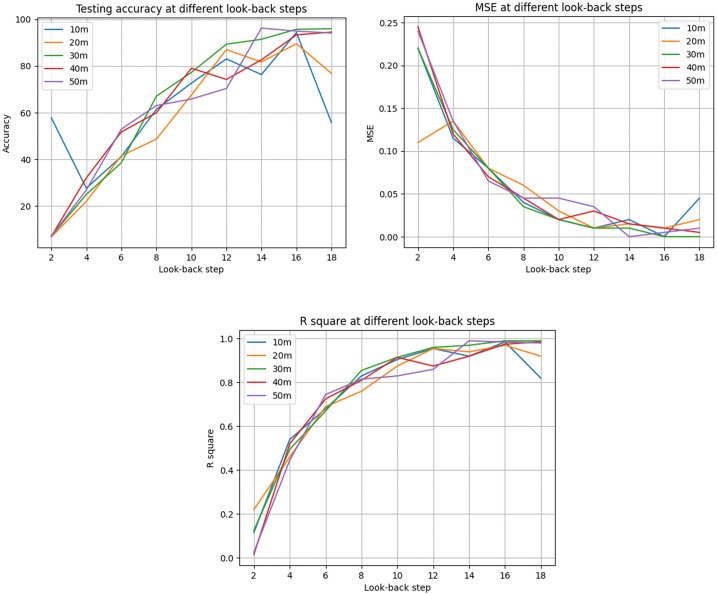
Testing accuracy, MSE, and *R*^2^ values with different look-back steps.

The graphs in Fig 11 describe the effect of look-back steps on the model’s prediction accuracy, *R*^2^ values, and MSE. Prediction accuracy seems to improve with an increase in the number of look-back steps, but with trade-offs in hardware memory requirements and processing time. The graph indicates that using a small number of look-back steps, ranging from 2 to 8, yielded poor prediction results (below 70%). This can be attributed to the insufficient information available for the model to effectively forecast future steps. Using longer sequences of more than 16 steps slightly improved prediction accuracy but significantly increased computational demands on memory storage and processing time. The results suggest that an optimal range for look-back steps lies between 10 and 16 (which reach above 80%). This range provided the model with sufficient data to generate accurate predictions while maintaining reasonable processing time and memory requirements. Based on these findings, 16 look-back steps were selected as a fixed parameter for the model in subsequent experiments.

### 5.3 Training convergence and stability

**Fig 12:**
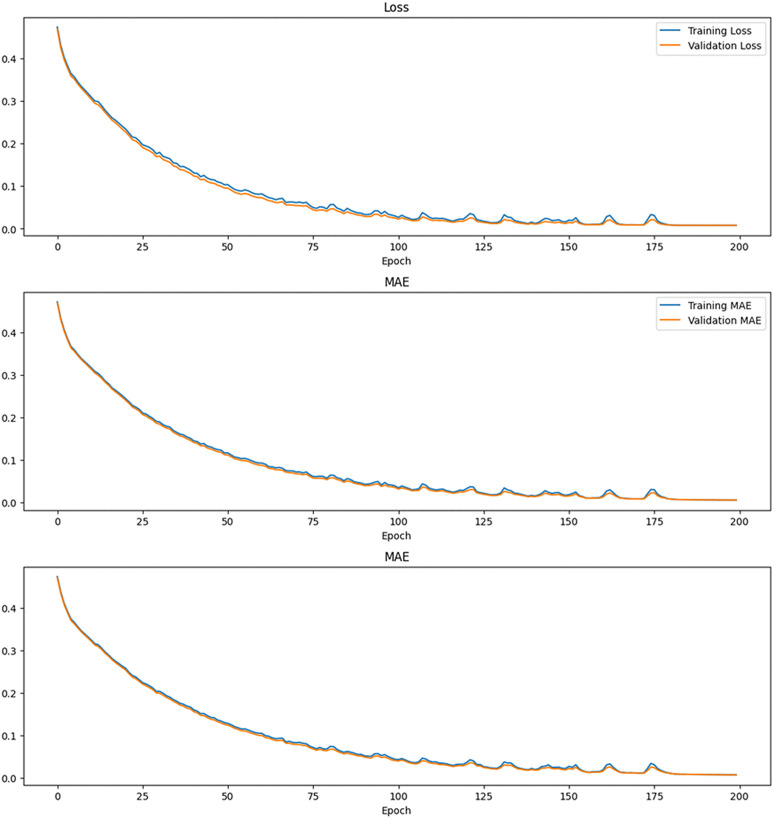
Training, validation loss, and MAE of two prediction steps in the case of 2-step prediction and 10m coverage radius.

Here we show the training and validation progress performed on the chosen AR-RNN using LSTM cells, as demonstrated in Fig 12:

Fig 12 shows the total loss of the model and also another type of error, the Mean Absolute Error (MAE) of the two steps after being trained and validated on the validation set at about 200 training epochs. The total training loss and the MAE of both training and validation phases converge at a low error score, close to “0,” showing that the model has achieved stability in learning and its potential can be used for prediction in the training phase. The other cases produced a quite similar trend of results, so the case of 2-step prediction in the 10m coverage radius network scenario is used to represent the convergence and stability of the model in each case.

### 5.4 Practical results

**Fig 13:**
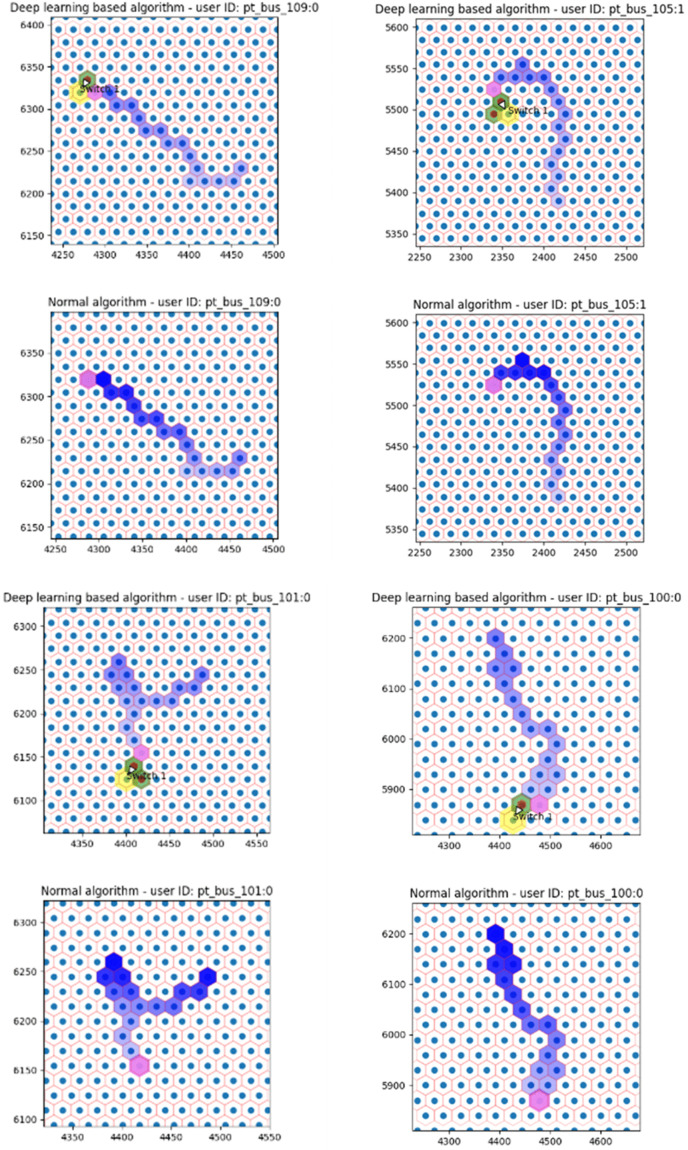
Illustration of practical prediction in the simulation scenario.

This subsection presents practical results, demonstrating how each UE operates on vehicles while employing the services within the configured network. The first result highlights the model’s capability in predicting the cells that a UE is likely to visit within the next *b* second(s) (Fig 13).

Fig 13 shows the cell-based mobility predictions of the UE as performed by the model. In the charts, the blue dots and the white-filled hexagons represent femtocells and their respective coverage areas within the network. The blue-filled hexagons indicate cells visited by the UE in the past, corresponding to the look-back steps. Purple hexagons indicate the current cells where the UE is located. Green hexagons represent the true future cells that the UE is predicted to visit, and the red dot inside each hexagon indicates the predicted cells of the model. If a red dot lies inside a green hexagon, the prediction is correct, and conversely, if a red dot lies outside a green hexagon, the prediction is incorrect. Yellow-filled hexagons indicate re-selected cells, which are chosen when the initially predicted cell is either barred or expected to experience transmission problems.

The scenarios depicted in Fig 13 highlight instances where the UE encountered barred cells in the upcoming moments. In the regular case (those with title: Normal algorithm), at the current moment (cells are colored in purple), since there is no prediction, the users will go to a barred cell and suffer disconnection at the next moment of each case. In the case of a mobility-resource prediction-based scheme (those with title: Deep learning based algorithm), at the current moment (cells are colored in purple), the system knows the cell the user will come to in the next moment, and since it is potentially barred, the system indicates another cell (yellow cells), preparing some possible selection and resource allocation processes and waiting for the user to come in the next moment. This enabled the system to preemptively select and allocate resources for a replacement cell. When the UE transitioned to the next moment, the pre-selected cell was already prepared to connect, thereby avoiding disconnection.

**Fig 14:**
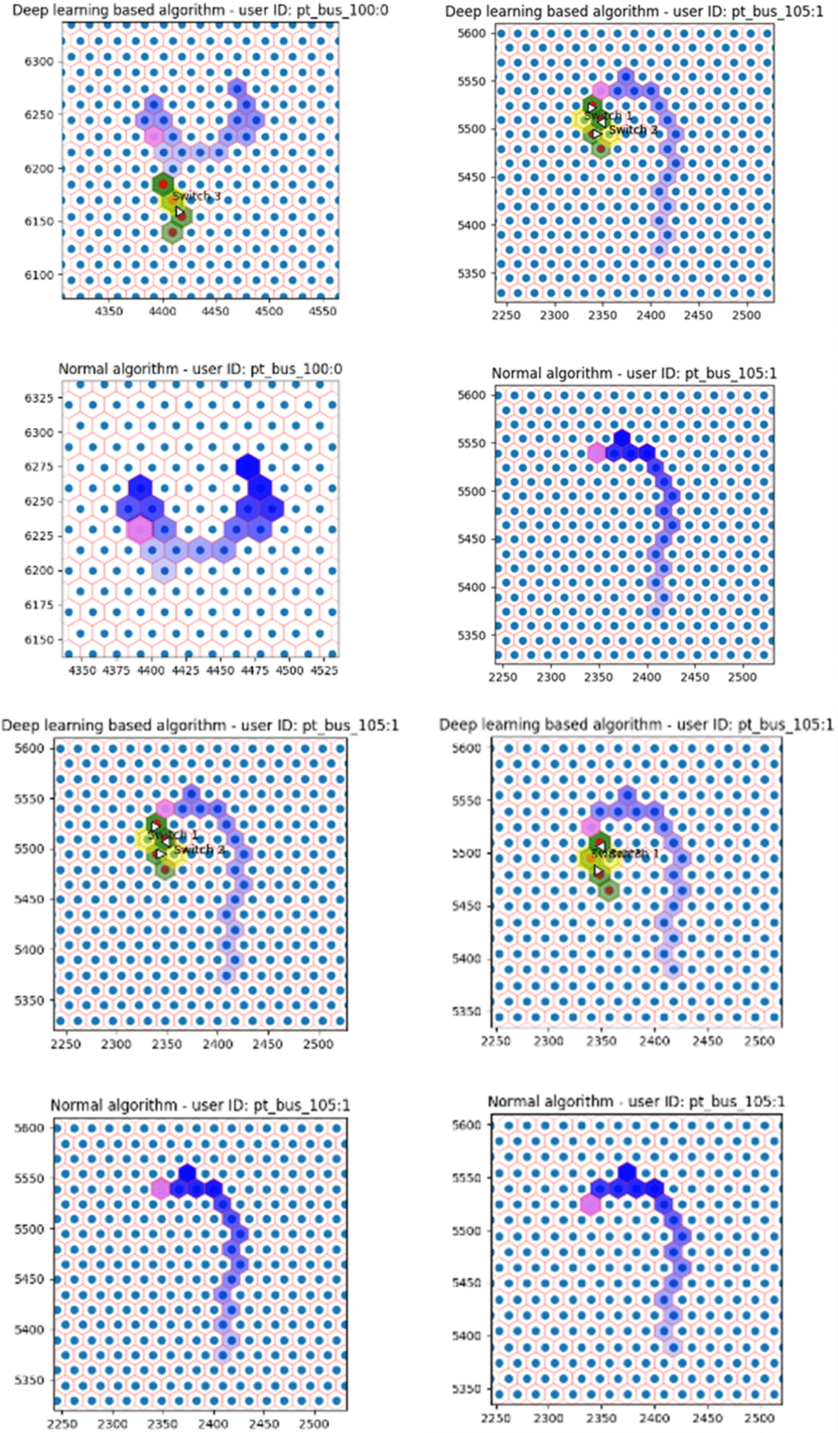
Illustration of practical multiple-step prediction in the simulation scenario.

Furthermore, Fig 14 provides an illustration of practical multiple-step predictions within this simulation scenario. Prediction of multiple steps ahead can benefit the system by identifying more cells that may experience barring and providing time to reassess the plausibility of these predictions according to UE behavior observed during transitions between cells. In practical applications, predictions for one or two steps ahead are sufficient for networks to evaluate potential cell barring. Predictions for three or more steps ahead, however, are generally useful only as supplementary information to aid the network in refining estimations of barred cells rather than directly influencing an incoming prediction. If a current prediction proves inaccurate, the network can still observe as the UE progresses to subsequent cells and then reevaluate the incorrect information according to the predictions for three or more steps ahead (by calculating the average accuracy over a series of predictions).

An analysis of user throughput reveals how the avoidance of potentially barred cells and the preparation of handover procedures in advance affect both the throughput and the latency in the connection between the UE and the network. From this perspective, the advantage of applying the proposed model over standard approaches (for example, schemes without mobility-resource-based prediction) can be demonstrated and understood.

**Fig 15:**
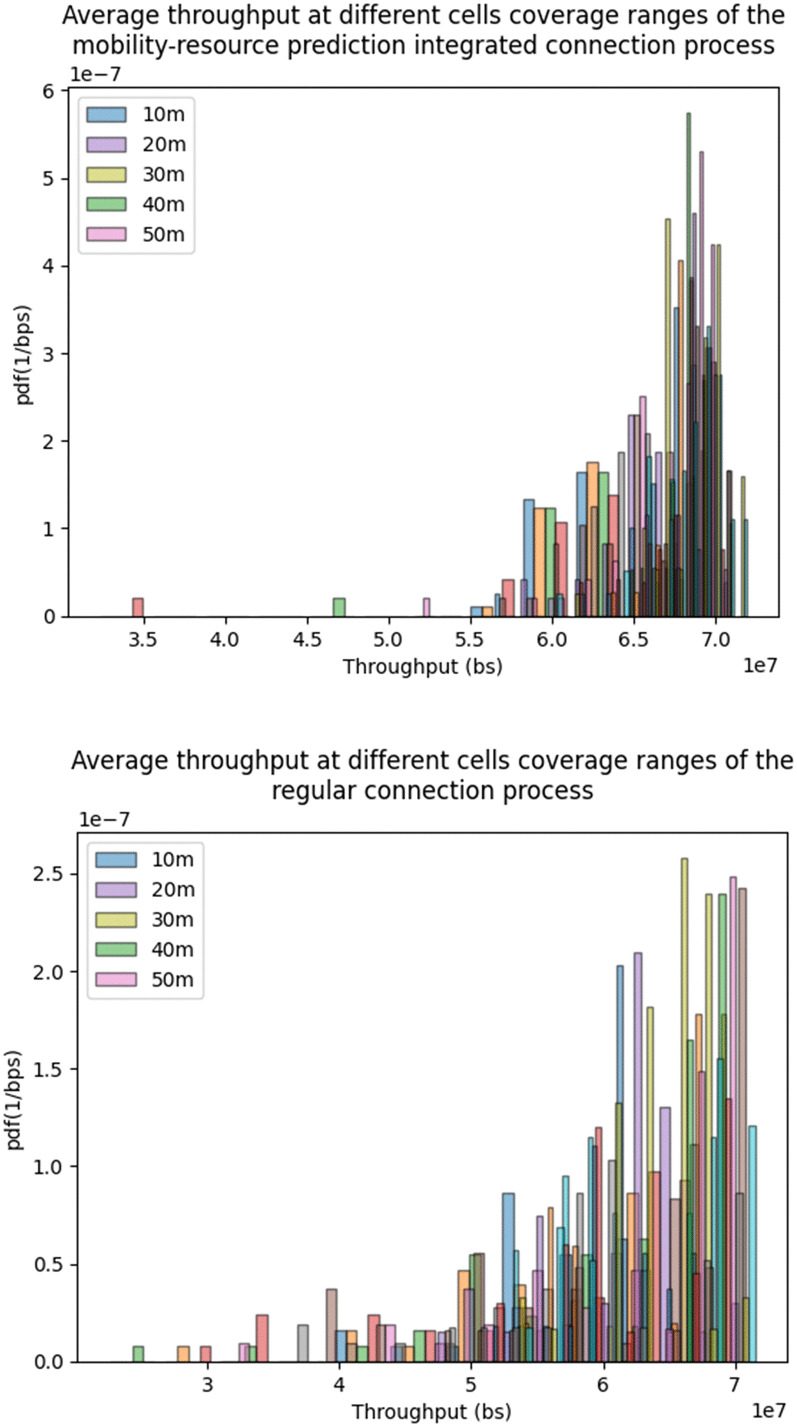
Average throughput of considered users between the proposed mobility-resource prediction scheme and the regular scheme.

Fig 15 graphically compares the average throughputs of the UE across five femtocell coverage radii under the proposed mobility-resource prediction scheme and the traditional standard scheme [55], respectively. The average throughputs have noticeable variations depending on the cell coverage range, clustering around 68 Mbps or 69 Mbps. Under the proposed scheme, the system’s average throughput peaked at approx. 65 Mbps or 66 Mbps, notably higher than the 3 Mbps maximum throughput under the standard scheme. Moreover, the proposed scheme seems to have less variation in throughput across different coverage ranges than the regular scheme, particularly at the 10m and 20m ranges.

**Fig 16:**
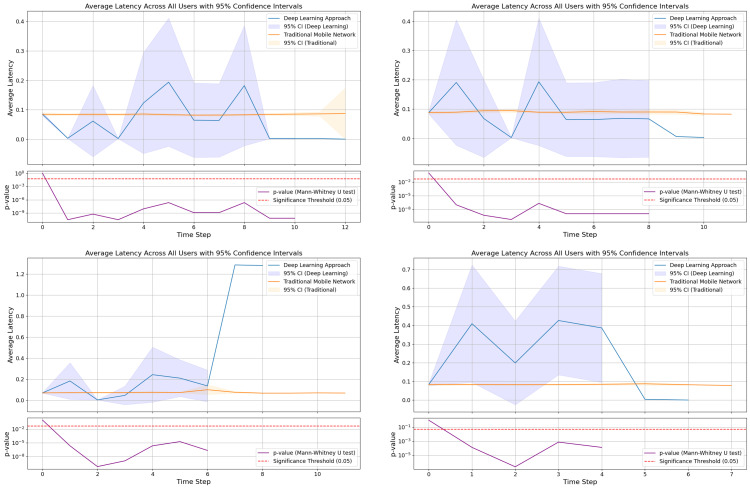
10-meter coverage radius.

**Fig 17:**
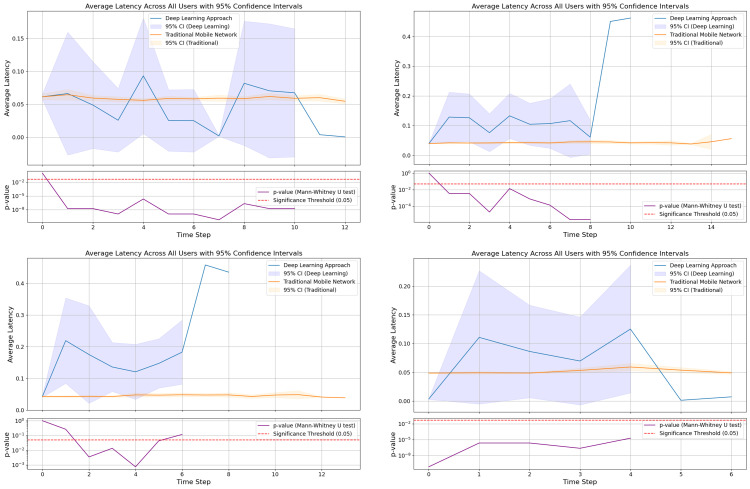
20-meter coverage radius.

**Fig 18:**
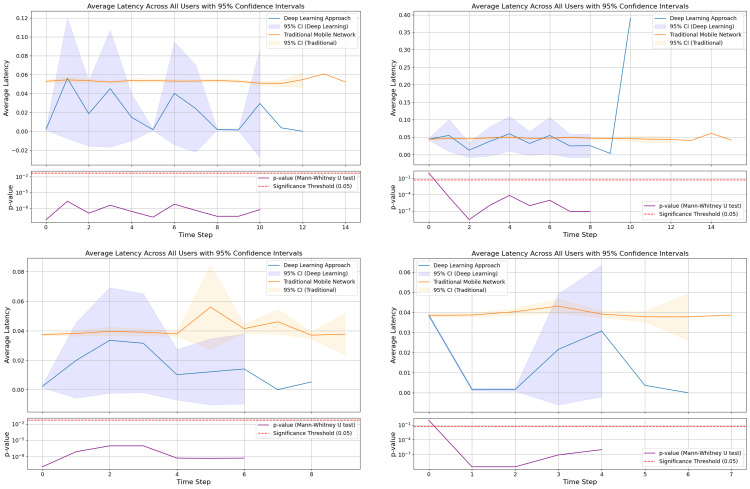
30-meter coverage radius.

**Fig 19:**
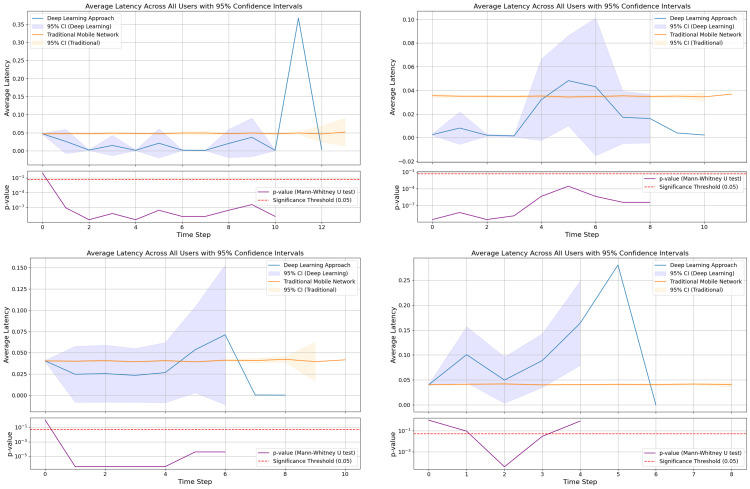
40-meter coverage radius.

**Fig 20:**
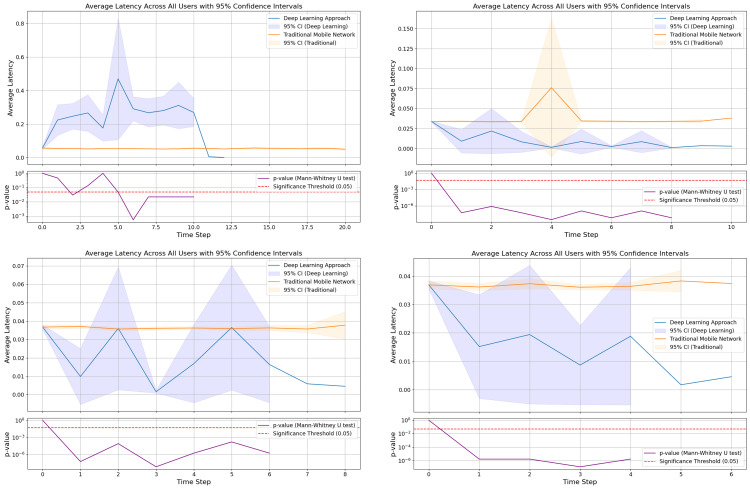
50-meter coverage radius.

Latency is another important metric in this system. It is the time required for the resource allocation to be performed. From Figs 16–20, showing the comparisons of average latency required for resource allocation under the standard scheme, both with and without mobility prediction for all users in 5 cases of the coverage range. From top left to bottom right of each case is the result of 1 step, 2 steps, 3 steps, and 4 steps ahead prediction, and below each result is its associated Mann-Whitney U Hypothesis testing at the 95% confidence level:

Initially, the average latency for all UEs in the two schemes was equivalent, as the proposed model required preparation time to generate predictions during the simulation. In practice, this delay occurs only once at the algorithm’s initialization and can be mitigated by configuring “warm-up” positions. The figures clearly show that when the proposed method commences predictions and initiates preparatory procedures, it achieves consistently lower average latencies than standard methods, especially when barred or resource-depleted cells are encountered.

To rigorously validate the performance improvement, we evaluated the statistical significance of the latency reduction achieved by the proposed predictive method relative to the traditional baseline using a formal Mann-Whitney U test. It is widely documented in telecommunications research that network latency does not follow a normal distribution, but rather exhibits a heavy-tailed distribution with significant outlier spikes caused by congestion and queuing [56]. Because parametric tests like the Student’s t-test are highly sensitive to such outliers, the non-parametric Mann-Whitney U test is the most suitable choice, as it is robust to non-normal network latency data [57]. The test confirmed that our proposed predictive approach yields a statistically significant improvement in latency (*p* < 0.05).

The bottom subplots in Figs 16–20 track the resulting *p*-values over time. For the vast majority of the active simulation duration, the *p*-value remains well below the 0.05 significance threshold (indicated by the red dashed line). Occasional mid-simulation spikes above this threshold correspond to the case of prediction errors (visible as sharp upward deviations in the blue latency curve), during which the system relies on the fallback mechanism. Overall, however, this provides strong statistical confirmation that the Deep Learning approach yields a significant reduction in latency compared to the traditional reactive network.

To provide robust statistical validation, we have included 95% confidence intervals (CI) in our latency comparisons, as shown from Figs 16–20. This analysis allows for an open discussion of both best-case and worst-case scenarios, directly revealing the performance variance of our approach. The traditional network consistently exhibits a narrow confidence interval, confirming its stable and predictable performance. In contrast, the deep learning approach shows a significantly wider confidence interval, which is highly informative. This variance can be understood by examining its two underlying components:

*Accurate Prediction Scenario:* In most handover events, underpinned by the high prediction accuracy, the mobility and resource predictions are correct. Potential cell barring or resource depletion is either correctly anticipated (allowing selection of a prepared alternative) or correctly predicted not to occur (allowing standard preparation for the target cell). In these successful prediction cases, the proactive preparations (resource precalculation, elimination of redundant cell selection, and bypassing barred cells) are highly effective. Consequently, the latency experienced by the UE drops significantly, approaching the minimal time required for connection establishment, observed in our results to be near 0.005 seconds (5 ms) or lower. This represents the optimal and convergent state achieved when the predictive framework operates as intended.

*Inaccurate Prediction Scenario:* In the less frequent instances where a prediction is inaccurate (for example, failing to anticipate barring, incorrectly predicting a problem, potentially due to insufficient historical data for specific rare patterns), the system must adapt. The proactive preparations might be misdirected, and the UE could face delays similar or even lower than the standard method when encountering an unexpected issue, requiring reactive measures such as selecting a different cell or waiting for resources. The latency in these cases would naturally be higher, contributing occasionally to upward deviations from the optimal 5ms latency within the overall average. Since our proposed idea here is mainly aimed to in-advance reserving the needed resources to mobile hosts during their movements inside a geographical area, covered by a cellular architecture, plus, the best feature for mitigation and recovery is currently not present in most of 5G network deployments as it is probably will be a standard for 6G communications, we only address the most simple and feasible approach to deal with wrong prediction: the “fallback mechanism,” in which the system will simply return to the traditional method to select the cell when the integrate/support mobility prediction fails. This, of course, will affect the latency as we mentioned above, as the system needs to run the traditional cell selection procedure after the prediction procedure. One other way to mitigate the wrong prediction is to use more advanced models/algorithms and/or train the models for a very long time with a vast amount of good-quality data. This approach obviously costs way more time and expense, as well as is more difficult to achieve in real life than just simply “falling back” to the traditional approach.

For more detail, in the worst case, the upper bound of the confidence interval, particularly at the sharp peaks of the graphs, reveals the high latency penalty incurred during inaccurate predictions. These latency spikes represent the worst-case performance, where the system must rely on its robust fallback mechanism (returning to reactive recovery), leading to higher latency. The overall average latency shown in the figures is therefore a composite of these two scenarios. Because our AR-RNN model maintains high prediction accuracy, the low-latency “best cases” are far more frequent than the high-latency “worst cases.” This explains why our system, on average, still achieves a consistent and substantial latency reduction compared to standard methods, resiliently handling occasional prediction errors without significant degradation of the average user experience.

It is important to note the behavior at the tail end of the time series in these figures (e.g., after time step 5 or 6). By this point in the simulation, most UEs have reached their destinations and completed their trajectories, leaving only a few active users in the network. When the sample size drops to a single user, it becomes mathematically impossible to compute a 95% confidence interval (which requires variance) or to perform a valid Mann-Whitney rank-sum comparison. Consequently, the shaded regions disappear and the *p*-value lines break. However, these terminal edge-cases are statistically insignificant compared to the broader trend. The stable and highly significant performance (*p* < 0.05) observed during the earlier, data-rich time steps—when the full cohort of users was active—serves as the definitive indicator of the system’s overall efficacy.

Finally, as anticipated, this performance volatility is exacerbated for longer prediction horizons. A comparison between the 1-step-ahead and 4-step-ahead prediction plots within each figure generally shows that the confidence interval for our approach becomes wider and the peaks more pronounced, directly correlating with the expected decrease in prediction accuracy. This once again shows the drawbacks of relying on the “fallback mechanism” of our proposed approach, which mitigates the wrong cell selection to guarantee the users reach the correct, working cells, at the cost of high latency. In the future, when a better mitigation technique is developed, this drawback can be effectively addressed, allowing our proposed prediction approach to clearly demonstrate its strength.

### 5.5 Outage probability performance

**Fig 21:**
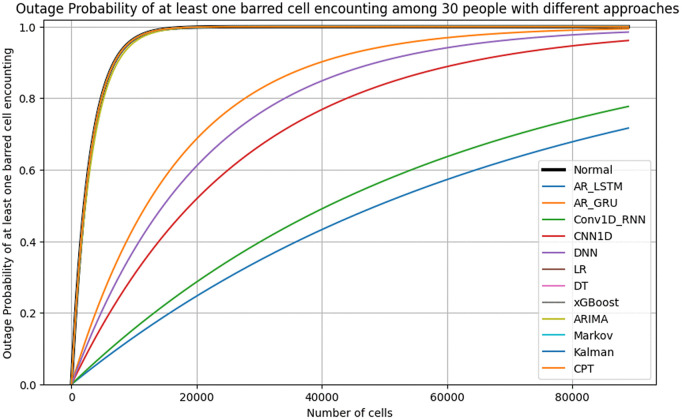
Network outage probability at 10 meters.

**Fig 22:**
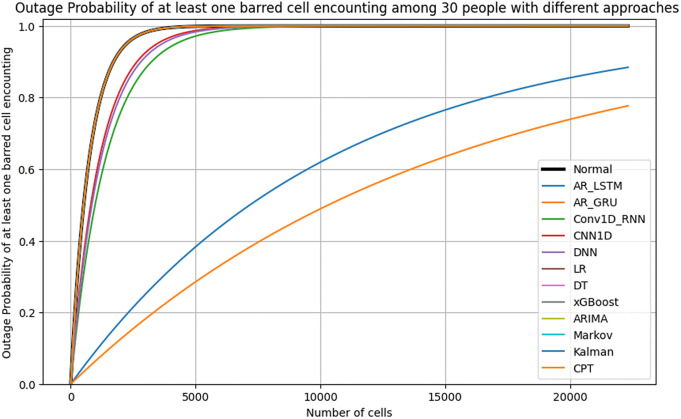
Network outage probability at 20 meters.

**Fig 23:**
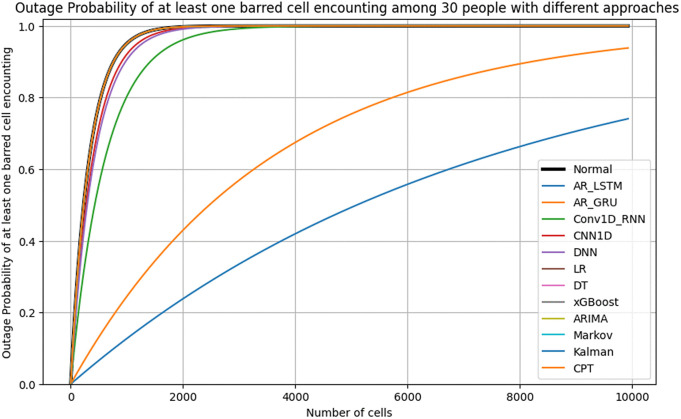
Network outage probability at 30 meters.

**Fig 24:**
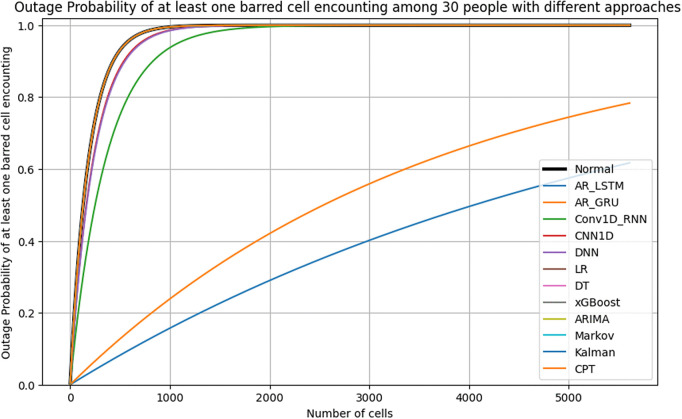
Network outage probability at 40 meters.

**Fig 25:**
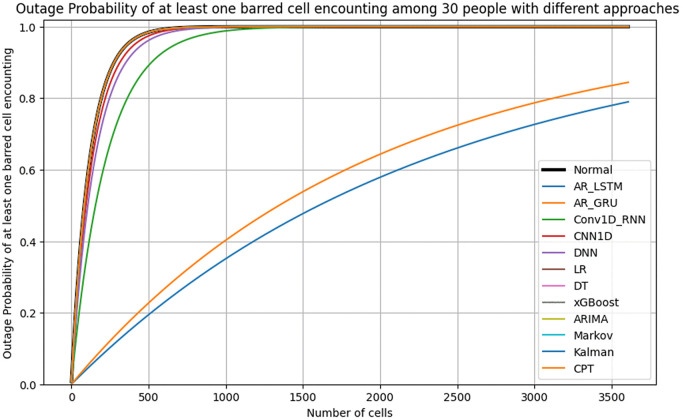
Network outage probability at 50 meters.

Outage probability is defined as the probability that at least one user encounters a barred cell within the entire considered network (modeled as a homogeneous network). This metric was evaluated under two scenarios: (1) using only the standard connection and resource allocation scheme, and (2) using the connection and resource allocation scheme with mobility and resource prediction. Mobility prediction in the latter scenario was implemented using various algorithms. The results of the outage probability are graphed in Figs 21–25.

The black curve in the graphs represents the outage probability under standard handover and resource allocation procedures. The other colored curves represent the network outage probabilities under the proposed model using different prediction algorithms. The results indicate that networks using the prediction method achieved lower outage probabilities than standard handover and resource allocation procedures, regardless of the cell radius. Among the predictive methods, the deep learning algorithms achieved the lowest outage probabilities, particularly AR-LSTM and AR-GRU.

Between these two models, AR-LSTM demonstrated a lower outage probability as the cell radius increased and generally outperformed AR-GRU in most cases, with the exception of the 20-meter cell radius. This suggests that AR-LSTM is more adept at handling complex scenarios than AR-GRU, providing an overall slightly higher performance.

In summary, these results highlight the advantages of using the proposed mobility-resource prediction approach in handover and resource allocation procedures. The approach offers improved performance in terms of maintaining high throughput, low latency, and reduced outage probability for next-generation networks.

## 6 Validation in HetNet and real-world mobility data

### 6.1 Roma taxi dataset and HetNet structure

To address the limitation of using purely simulated data and to test the generalization of our proposed AR-RNN model, in this section, we conducted an extensive real-world validation using a public mobility data set and a HetNet structure.

**Fig 26:**
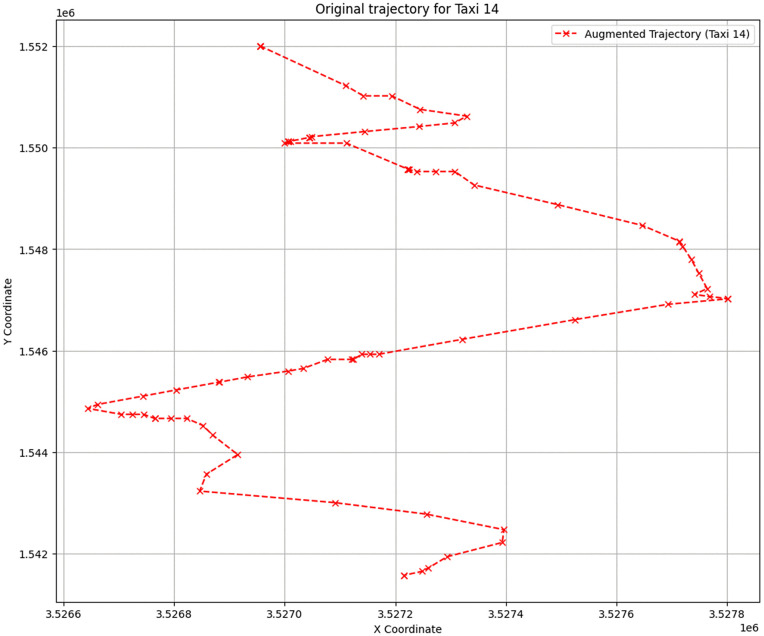
The illustration of a vehicle’s trajectory in the Roma/taxi/taxicabs dataset.

The data set we proceed to use is the Roma/taxi/taxicabs dataset [58]. This dataset contains GPS trajectories of about 316 taxis operating in Rome, Italy, and provides a realistic representation of urban vehicular mobility patterns. Fig 26 presents a user’s movement record in the data set.

**Fig 27:**
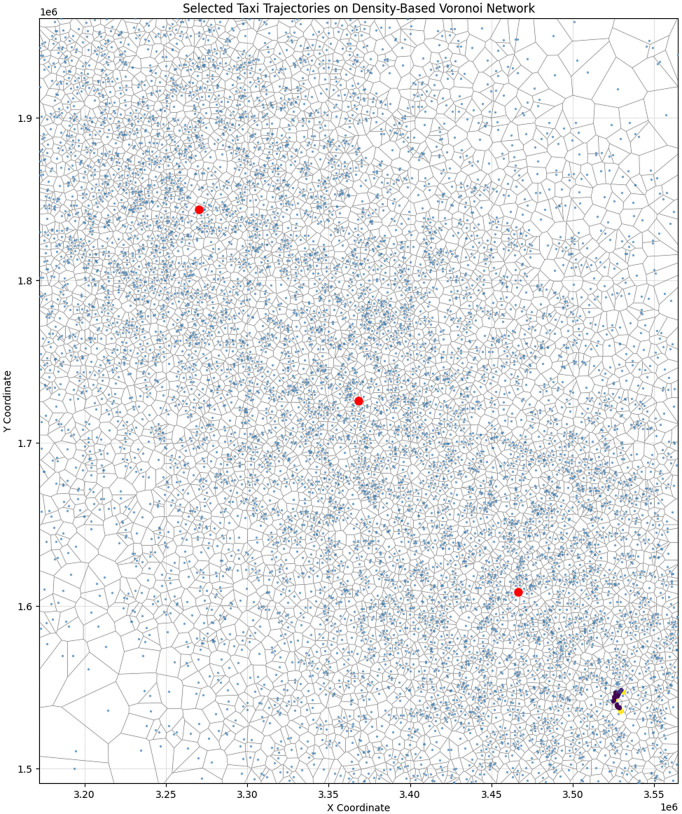
The illustration of the HetNet used in the experiment.

For the non-homogeneous network system, a Voronoi network topology is generated using a density-based Voronoi tessellation to represent the HetNet to overlay on the users’ trajectories data and create the experiment scenario, as illustrated in Fig 27. This approach creates an irregular cell layout that more closely mirrors real-world network deployments, with smaller cells in denser areas. In this comprehensive HetNet evaluation, we focused on modeling the topological heterogeneity of the network. To isolate the impact of this irregular topology and variable cell traversal times on the prediction algorithm’s performance, we maintained uniform transmission parameters across the network. This allows us to verify the model’s adaptability to non-uniform spatial structures without the confounding variables of heterogeneous signal configurations.

In Fig 27, each Voronoi cell represents a coverage area of the BSs, which are the centers of these Voronoi cells. Similar to the main experiment, this network is overlaid on the Roma/taxi/taxicabs dataset, and based on that, we transform these continuous GPS coordinates into a discrete cellular structure for our model. We applied our data processing pipeline to convert these trajectories into sequences of cell IDs, as done in our main simulation.

All models and algorithms have inherent limits, and the RNN with LSTM is no exception. So, to manage the scale and diversity of the 316 unique users in the dataset, we adopted a partitioned, multi-model approach. The users were divided into 11 distinct groups based on their identifiers, and a separate AR-RNN model was trained for each group. This strategy enables parallel training and allows each model to specialize in the specific mobility patterns of its assigned user cohort. Similar to the former experiment of the Homogeneous network in Singapore city, the experiment on HetNet in Rome, Italy, is performed with a 16-step look-back, in the cases of from 1 to 4 step-ahead prediction. The experiment contains a barred cell trigger event and random interfering users when the considered user comes to a cell. The experimental results for throughput, latency, and outage probability are presented in the following section.

### 6.2 Experimental result

#### 6.2.1 Practical prediction.

**Fig 28:**
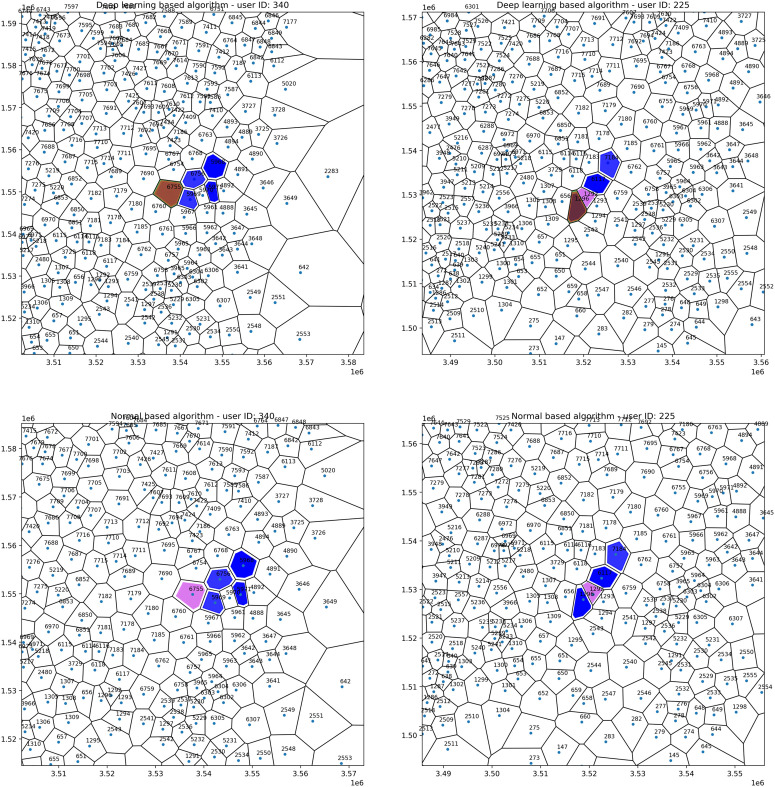
The prediction performed on user 340 and user 225. No barred cell encountered.

**Fig 29:**
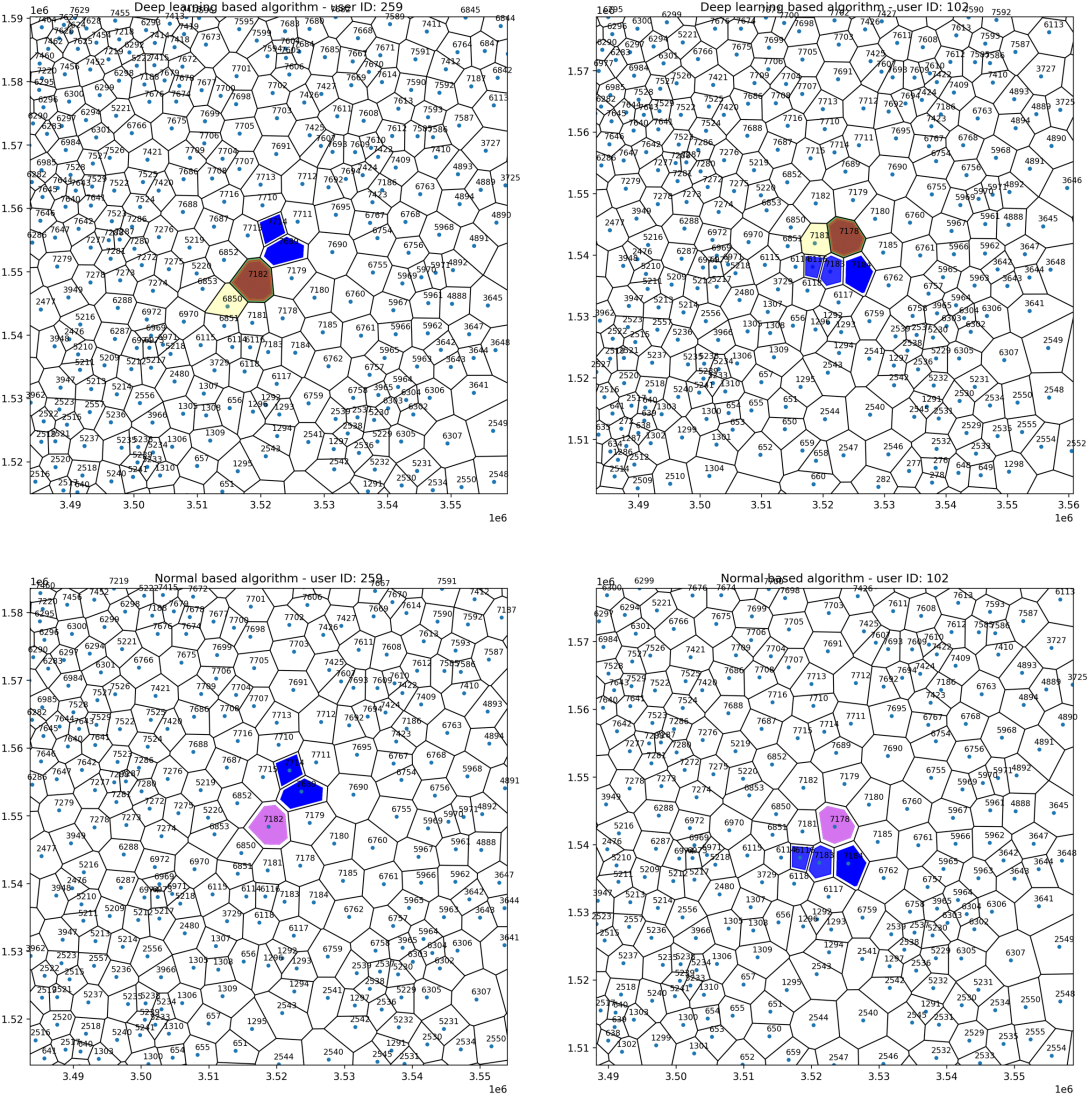
The prediction performed on user 259 and user 102. Barred cells happen.

**Fig 30:**
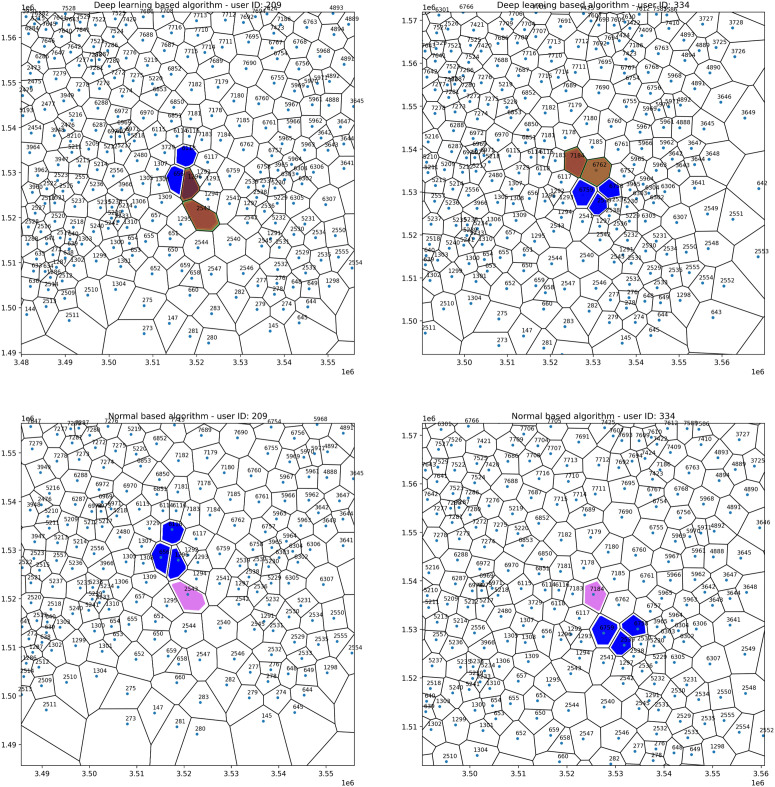
The prediction performed on user 209 and user 334, barred cells did not happen.

**Fig 31:**
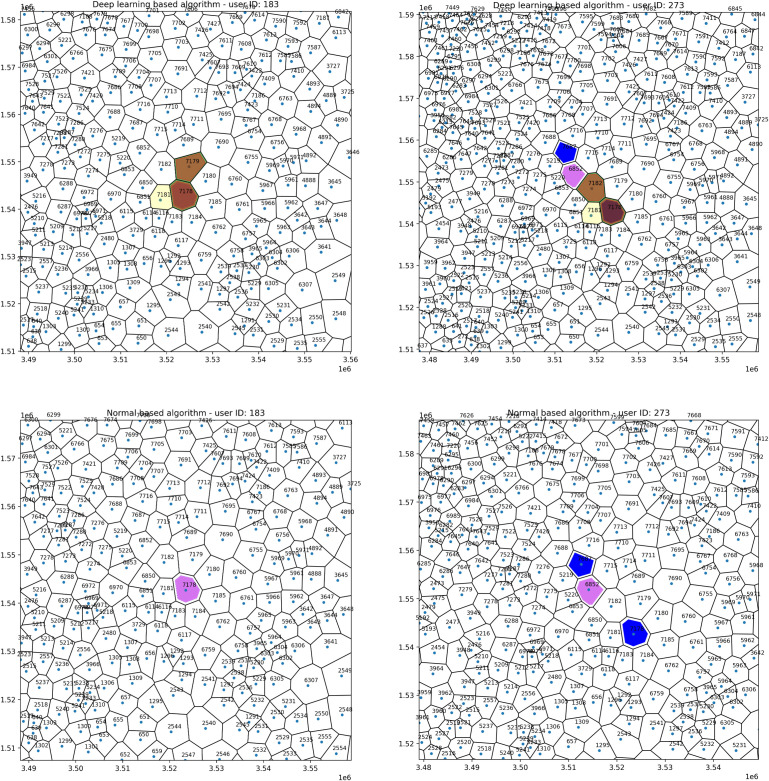
The prediction performed on user 183 and user 273, the case of barred cells happened.

**Fig 32:**
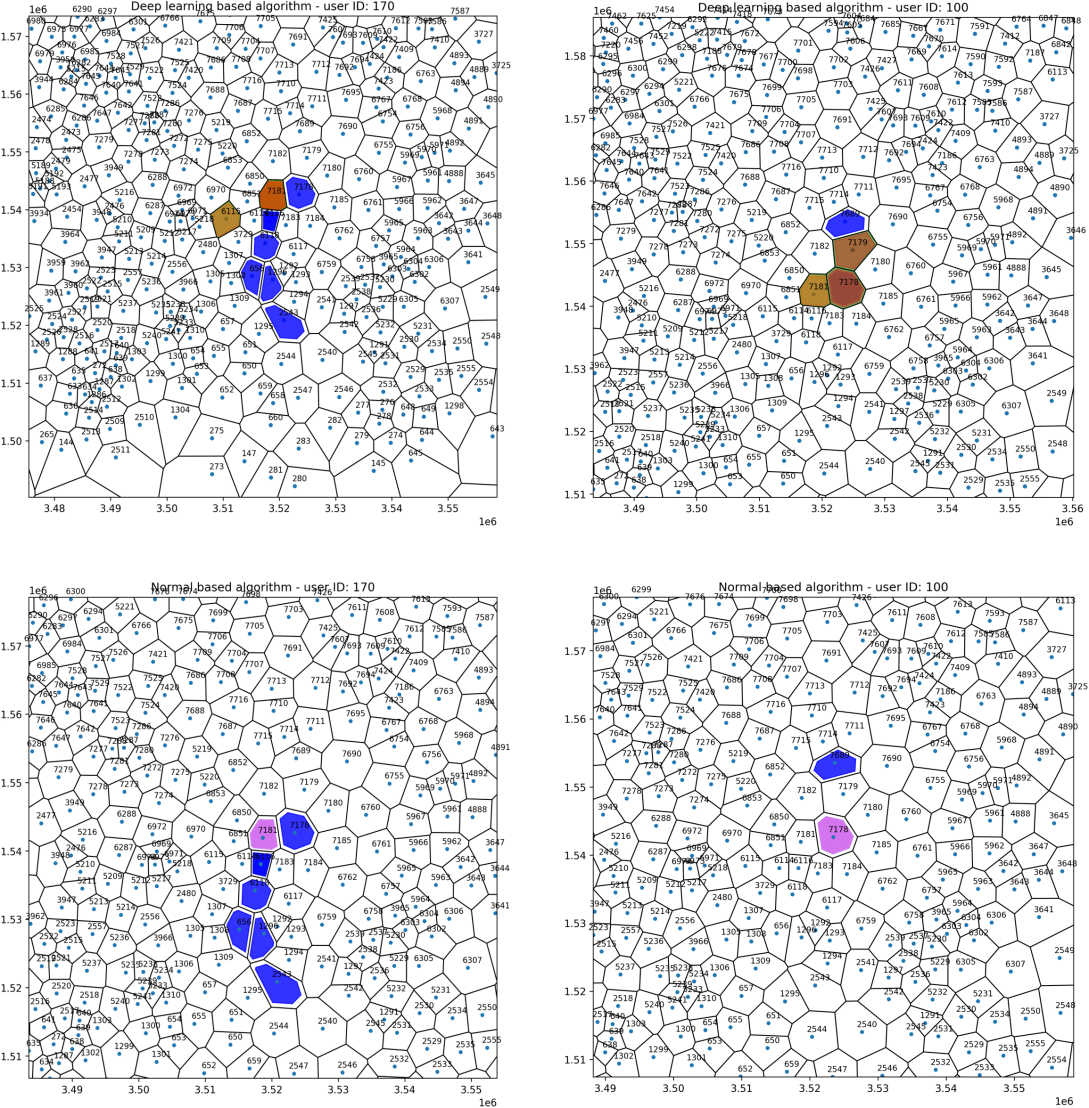
The prediction performed on user 170 and user 100. No barred cell occurs.

**Fig 33:**
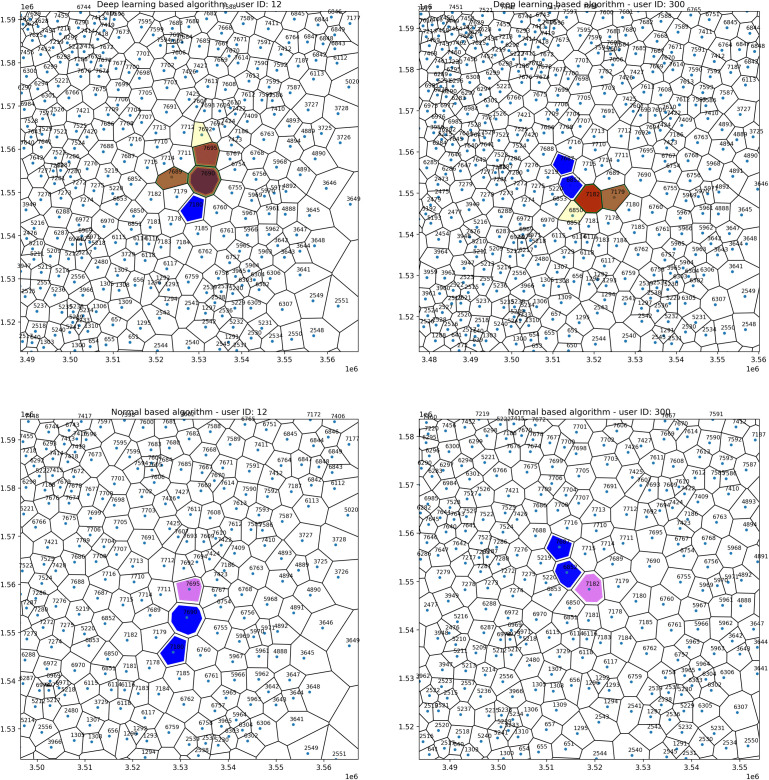
The prediction performed on user 12 and user 300, with barred cells occurs.

**Fig 34:**
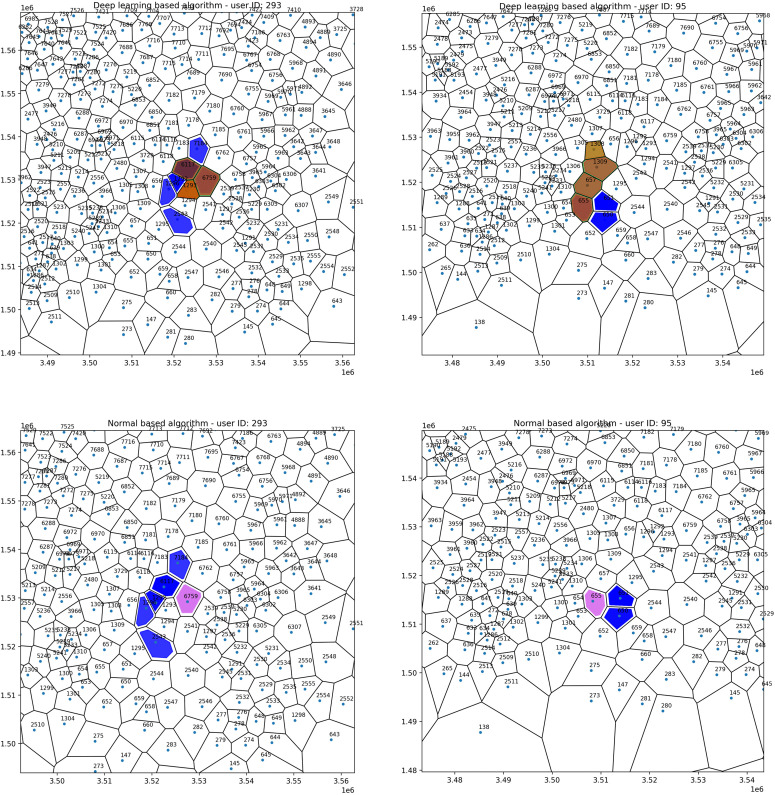
The prediction performed on user 293 and user 95. No barred cell encountered.

**Fig 35:**
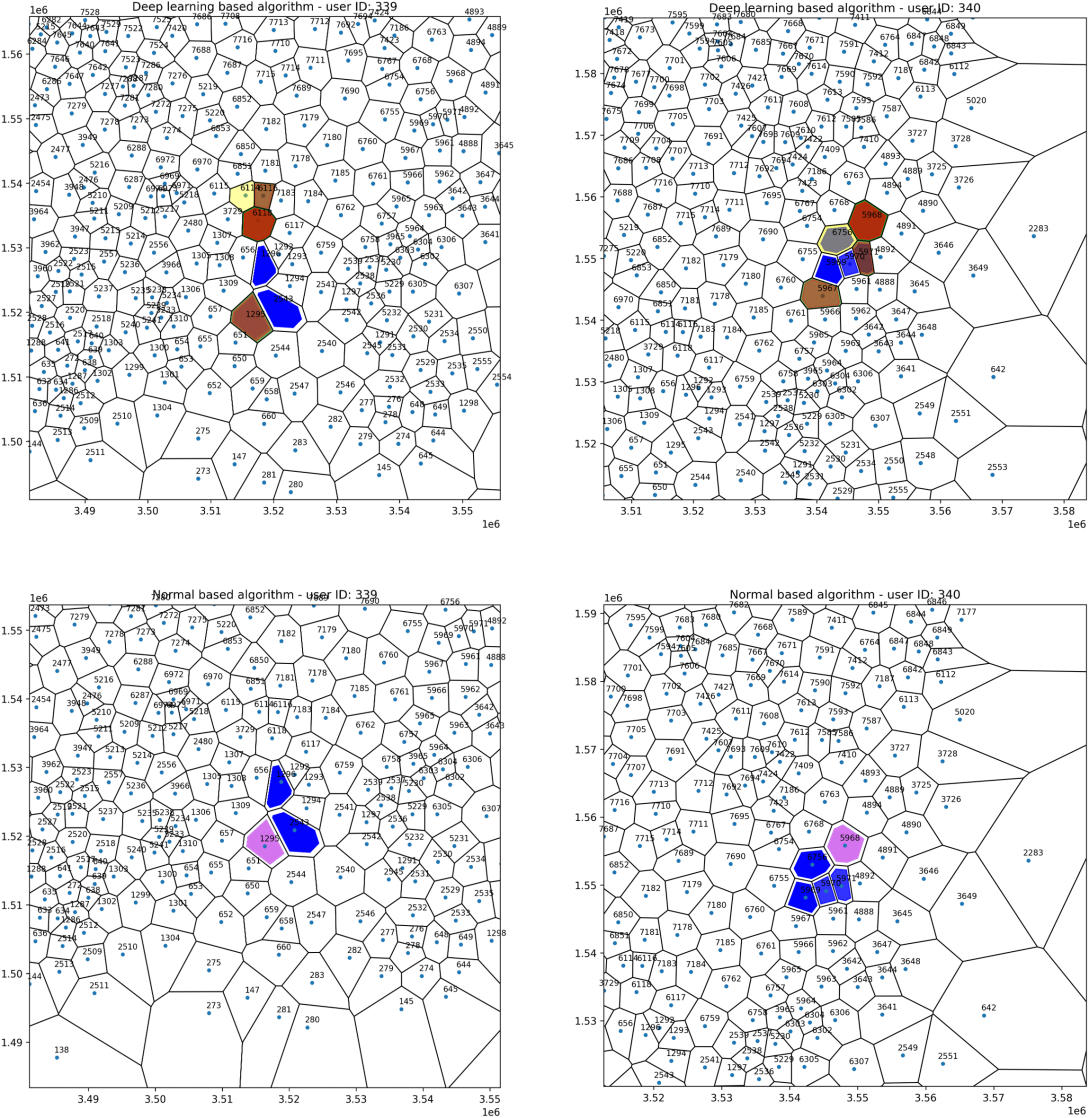
The prediction performed on user 339 and user 340. Barred cells encountered.

The following figures from Fig 28–35 show the prediction of our proposed scheme’s algorithm and the traditional algorithm performed on the users’ testing samples. For detailed observation clarification, cells that are colored in blue represent the past cells the users visited before, purple cells represent the current cell the users are staying in, cells that are colored in green are the actual cells the users are going to arrive in the near future, and red cells indicate the cells predicted by our proposed algorithm. If the red cells lie within the green cells (in this HetNet experiment, when overlapping, they might form brown cells), then the prediction is correct. The situation where purple cells, or any other colored cells, are overlapped with different colors indicates that the user stays within a cell for a long time or their route passes by them several times. The cells colored in light yellow are the replaced cells that the algorithm plans for the user if the predicted cell is suspected of becoming barred/problematic at the moment the user arrives. Each result is shown in pairs: the upper graph shows the Deep Learning mobility and resource prediction algorithm, and the lower graph shows the Traditional algorithm.

**One-step-ahead prediction:**

Fig 28 illustrates the model’s performance for the immediate next time step. In these scenarios, the high accuracy of the AR-RNN is evident as the predicted red cells perfectly overlap with the actual green cells, forming a brown indicator. This prediction allows the network to prepare handover resources with high confidence, minimizing latency for the immediate transition.

When barred cells potentially occur at the predicted cells, as shown in the situation of user 259 and user 102 in Fig 29, the proposed algorithm right away scans all the cells around them to choose the best cells for replacement and prepares that cell to be assigned to the user right away, the moment they come. The traditional algorithm cannot select the “yellow” cells for the users due to the lack of prediction.

**Two-step-ahead prediction:**

Figs 30 and 31 extend the prediction on the users to two steps. Here, we observe the system’s proactive management capability. When the predicted target cell is flagged as problematic, the system activates the steering mechanism to select replacement cells, as in the 1-step-ahead prediction case, but for two future time steps. This demonstrates the algorithm’s ability to not only predict mobility but also successfully bypass future network bottlenecks before the user encounters them. Making it two-fold more effective compared to the traditional approach.

**Three-step-ahead prediction:**

As the prediction horizon increases to three steps in Figs 32 and 33, the trajectory complexity grows. The figures demonstrate the model’s robustness in maintaining trajectory alignment even as the user moves further from the current location. The comparison with the traditional method (lower graphs) highlights the advantage of the proposed scheme: while the traditional method reacts only upon arrival, our scheme reserves the necessary resources in the correct (Brown) or alternative (yellow) cells well in advance.

**Four-step-ahead prediction:**

The Fig 34 and 35 represent the most challenging scenario with a four-step prediction. While the uncertainty of user movement naturally increases over longer timeframes, the model continues to provide valuable directional guidance. Notice the case of the user 340, the user is currently at cell 5968, that cell would get barred at the next moment, and the model predicted correctly that the user would still stay in that cell at that moment, it scanned and saw that the cell 6756 as the best replacement cell for the user of all surrounding cells (6768, 6763, 4894, 4891, 4892, 5971) and since the user recently visited cell 6756 in the past moment before coming to cell 5968, the connection would be maintained at the cell 6756, avoiding disruption as much as possible until they go too far away from it or until a better one available. It shows that even though the cell serving the user is currently good, the algorithm always actively predicts the next movement of the user so that if there is any sign that the serving cell will get barred, the algorithm will immediately find an alternative cell to prepare to connect with the user in the next moment the serving cell gets barred.

In scenarios where the network operates without cell barring or resource depletion, the prevalence of brown hexagons (formed by the overlap of predicted Red and actual Green cells) confirms the high spatial accuracy of the AR-RNN model. Despite the complex, non-linear nature of the real-world taxi trajectories and the irregular shapes of the Voronoi cells, the model successfully captures the underlying movement patterns. This precise forecasting allows the network to pre-allocate resources at the correct target cell, thereby reducing the signaling overhead and connection setup time compared to the reactive approach shown in the lower graphs.

The distinct advantage of the proposed scheme is visualized by the light yellow hexagons. These appear when the model correctly predicts the user’s trajectory but identifies the target cell as problematic (barred or congested). In these instances, the system does not merely predict the user’s location; it actively intervenes. By steering the user to a pre-selected alternative (yellow cells) rather than the failing target, the algorithm prevents the user from entering a “dead zone.” This visualization confirms that the system effectively translates predictive intelligence into actionable network decisions, maintaining connectivity in situations where the traditional algorithm would likely suffer a handover failure or significant service interruption. On the other hand, the Traditional algorithm, without any prediction or estimation of future events, has no means to prepare for replacement (yellowed cell) until the users arrive at the cell, whether or not the monitoring system detects potential barred cells. This will definitely cause disruption in service.

#### 6.2.2 Users’ average throughput.

**Fig 36:**
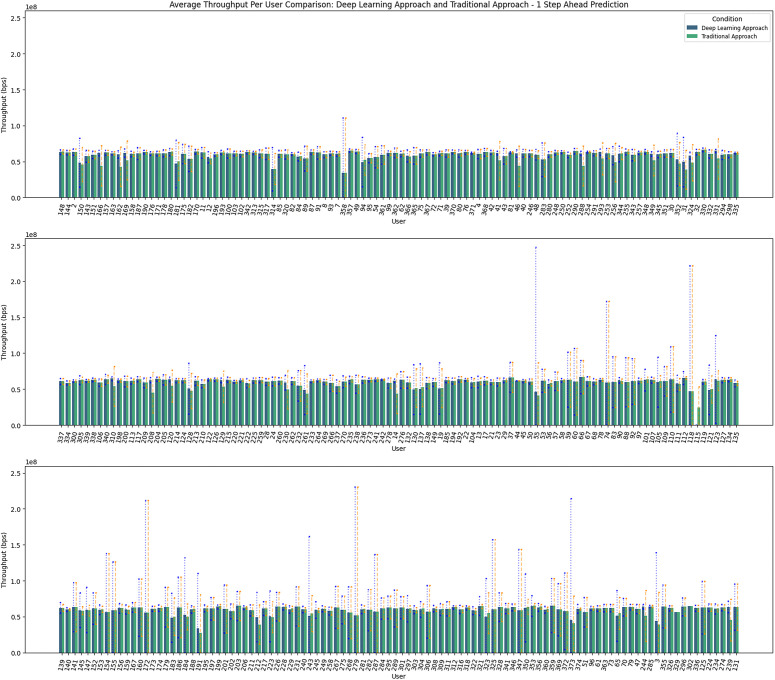
The throughput comparison per user (mean of transport) on the Roma/taxi dataset with 95% confidence intervals in the cases of 1-step-ahead prediction.

**Fig 37:**
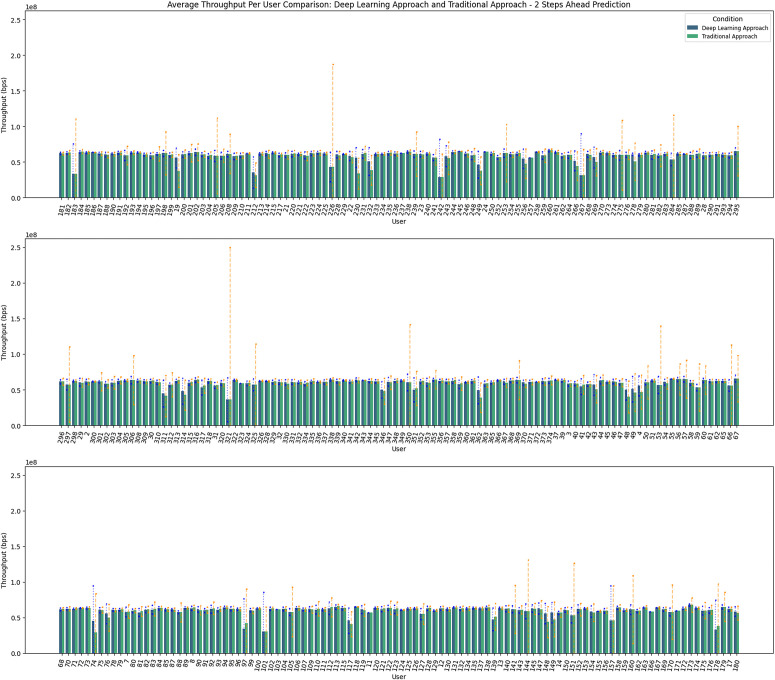
The throughput comparison per user (mean of transport) on the Roma/taxi dataset with 95% confidence intervals in the case of 2-step-ahead prediction.

**Fig 38:**
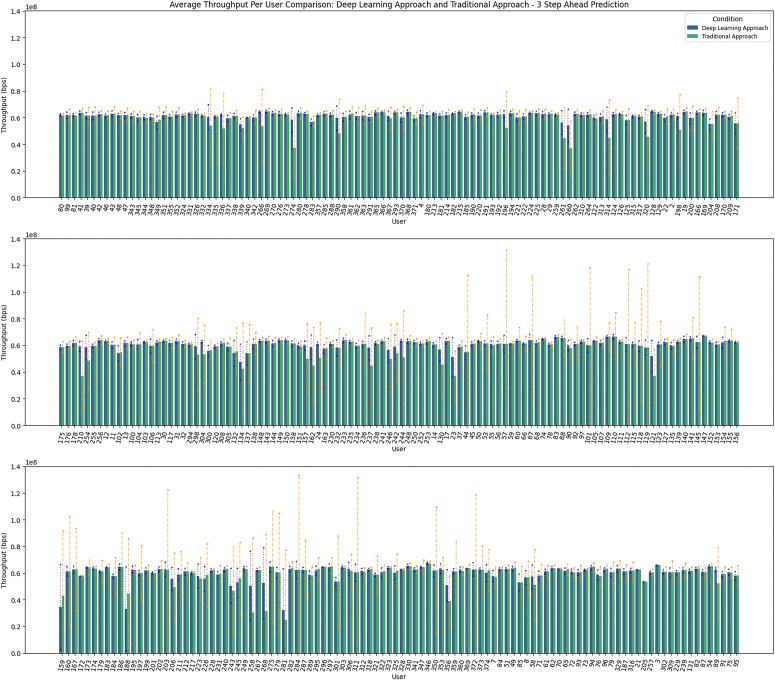
The throughput comparison per user (mean of transport) on the Roma/taxi dataset with 95% confidence intervals in the case of 3-step-ahead prediction.

**Fig 39:**
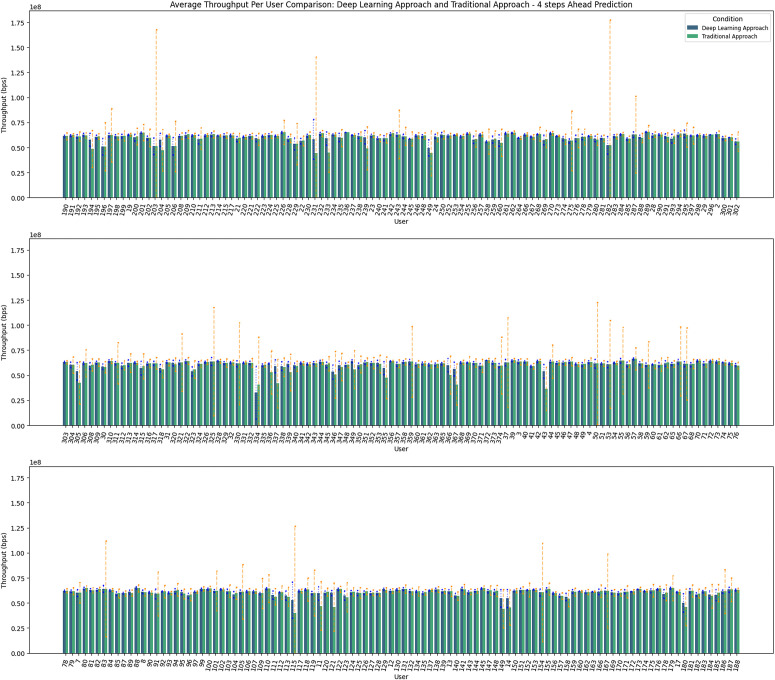
The throughput comparison per user (mean of transport) on the Roma/taxi dataset with 95% confidence intervals in the case of 4-step-ahead prediction.

The figures from Figs 36–39 present a detailed per-user comparison of average throughput between the Deep Learning approach (blue bars) and the Traditional approach (green bars) across prediction horizons of 1–4 steps. Due to space constraints, the bar graphs are divided into 3 parts to cover all 316 users. Several key patterns emerge from this analysis:

a) *Baseline Stability vs. Predictive Volatility:* The most noticeable distinction is the consistency of the two approaches. The Traditional approach (Green) maintains a relatively uniform throughput profile across most users. This reflects the reactive nature of standard handover protocols—they are “safe” and predictable but do not maximize potential resource utilization. In contrast, the Deep Learning approach (Blue) exhibits high variance, characterized by significant peaks and deep troughs.
b) *The “High-Reward” Scenario:* In some cases of users, particularly visible in the 1-step and 2-step predictions (Figs 36 and 37), the Deep Learning approach significantly outperforms the traditional baseline. These peaks represent successful predictive handovers. In these cases, the model correctly anticipated the user’s movement and pre-allocated resources at the optimal target cell. This eliminated the signaling overhead and interruption time associated with reactive handovers, allowing the user to maintain a higher data rate. This validates the theoretical advantage of the proactive framework when the model is accurate.
c) *The Cost of Prediction Errors:* Conversely, there are specific users for whom the throughput of the predictive model drops significantly below the baseline or approaches zero. These instances correspond to prediction errors on the complex, noisy real-world dataset. When the model targets the wrong cell, the handover fails or is delayed while the system recovers. This results in a temporary loss of connectivity, which drags down the average throughput for that specific user. The wide 95% confidence intervals (orange dotted lines) associated with the blue bars quantify this uncertainty.
d) *Impact of Prediction Horizon:* Comparing Fig 36 (1-step) with Figure Fig 39 (4-step), we observe that the volatility subtly increases with the time horizon. While the model continues to achieve high throughput for users with predictable trajectories, the frequency of lower-throughput outliers increases in the 4-step scenario. This is consistent with the inherent difficulty of forecasting long-term mobility in a chaotic urban environment.

These results demonstrate that the AR-RNN model is not a magic algorithm that improves performance for every user at every time step under limited training conditions. Instead, it acts as a high-performance engine that requires a safety net. The potential for superior throughput is clearly demonstrated, but the variance underscores the necessity of the fallback mechanism (discussed in the Purpose and Operational Role subsection) to smooth out the troughs and ensure that users suffering from prediction errors can quickly revert to the stable performance of the traditional network.

#### 6.2.3 Users’ average latency.

**Fig 40:**
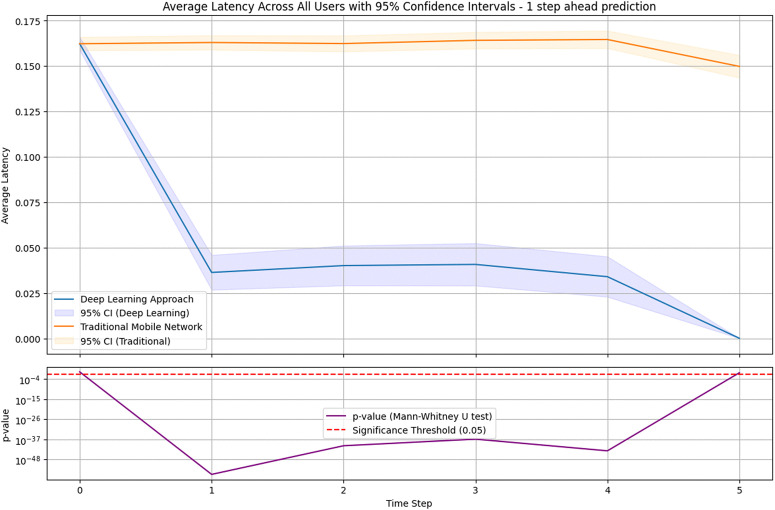
The average latency of all users between the proposed approach and the traditional approach in the case of 1 step ahead prediction.

**Fig 41:**
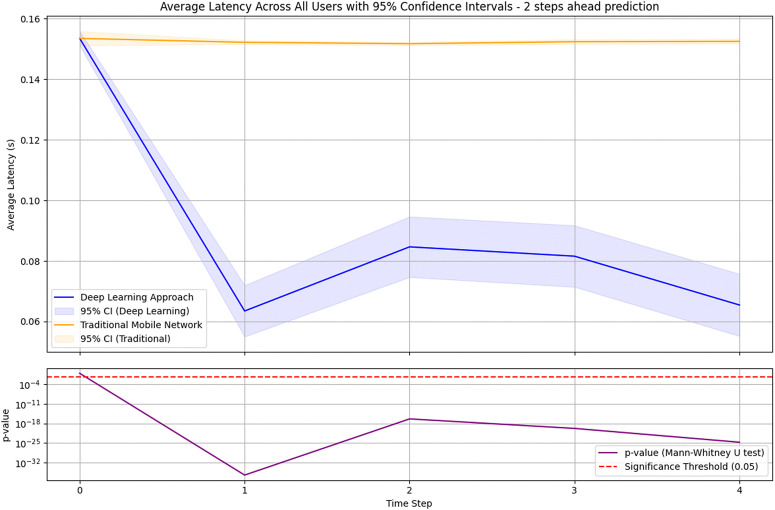
The average latency of all users between the proposed approach and the traditional approach in the case of 2-step-ahead prediction.

**Fig 42:**
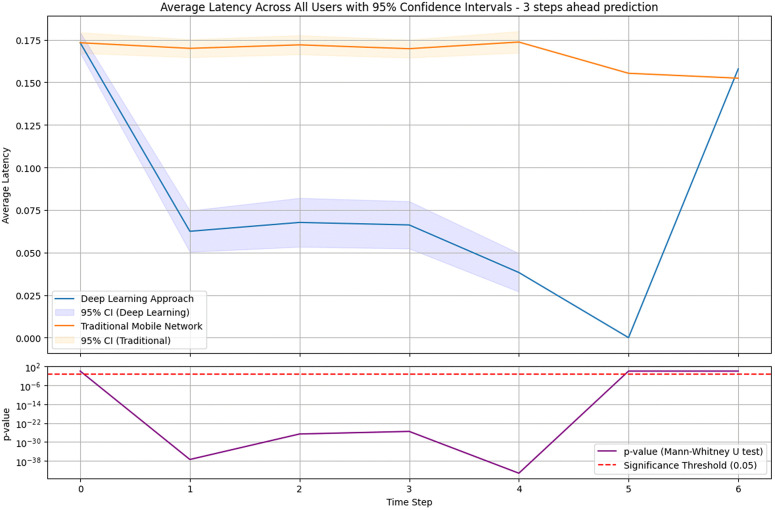
The average latency of all users between the proposed approach and the traditional approach in the case of 3-step-ahead prediction.

**Fig 43:**
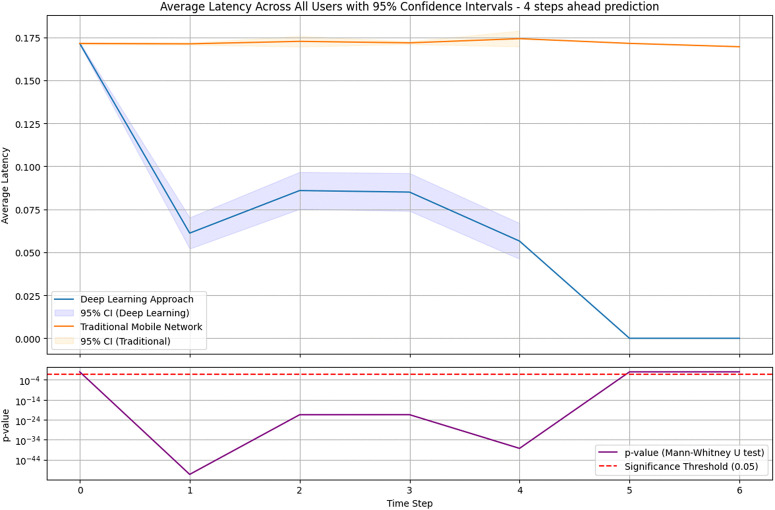
The average latency of all users between the proposed approach and the traditional approach in the case of 4-step-ahead prediction.

Figs 40–43 present the average latency across all users when evaluated under both the traditional approach and the proposed mobility/resource prediction approach. Similar to the Homogeneous experiment, the figures include 95% confidence intervals (shaded regions) and per-time-step *p*-values derived from a non-parametric Mann-Whitney U test (bottom subplots) for each result.

According to the figures from Figs 40–43, the results highlight four critical findings:

a) *Substantial Latency Reduction (which is the “proactive advantage”):* The most immediate observation is the dramatic drop in latency for the Deep Learning approach (blue line) immediately after the initial time step. While the Traditional approach (orange line) remains stagnant at approximately 0.16s, the proactive scheme consistently achieves latencies in the range of 0.04s to 0.08s, and sometimes can reach close to 0s (about 0.005s), as shown in Figs 40, 42, and 43, if the first best cell the algorithm scans is good and available right away. This confirms that when the AR-RNN correctly predicts the next cell, the proactive pre-allocation of resources eliminates the standard signaling overhead, resulting in a latency reduction of approximately 50% to 75% compared to the reactive baseline.
b) *Variance and Confidence Intervals:* Consistent with the throughput analysis, the Traditional approach exhibits a very narrow confidence interval, indicating high predictability but lower performance. In contrast, the Deep Learning approach shows a wider shaded region (95% CI). This reflects the mixed performance inherent to processing a chaotic real-world dataset: while most handovers are seamless (driving latency down), prediction errors cause occasional spikes. The width of this interval underscores the absolute necessity of the system’s fallback mechanism to handle these variance cases.
c) *Statistical Significance Validation:* To formalize these observations, the bottom subplots track the *p*-value over time. Throughout the active, data-rich periods of the simulation (e.g., time steps 1–4), the *p*-value curve remains firmly below the 0.05 significance threshold (indicated by the red dashed line). This mathematically confirms that the latency reduction achieved by the proposed approach in this complex HetNet scenario is statistically significant and not a random artifact.
d) *Impact of Trajectory Termination:* Similar to the homogeneous network experiment in the Practical results subsection, it is important to note the mathematical behavior at the tail end of the time series (for example, after time step 5 in Figs 42 and 43). By this stage, most users have reached their destinations, leaving only 1 or 2 active users. Consequently, statistical aggregation is no longer possible. Without sufficient comparative samples to compute variance or perform a rank-sum test, the confidence intervals disappear, and the *p*-value line spikes upward, losing statistical significance (crossing above the 0.05 threshold). Because these terminal points reflect the isolated behavior of a single user rather than a population average, the stable and highly significant performance (*p* < 0.05) observed in the earlier, data-rich time steps serves as the definitive indicator of the system’s efficacy.

These figures validate that the proposed scheme successfully breaks the latency floor of traditional methods. While prediction errors introduce variance, the average performance is significantly faster, proving that proactive management is key to enabling ultra-low latency applications in 5G/6G networks.

Ultimately, both the latency and throughput results from this real-world experiment tell the same critical story: while the predictive model holds great promise, its practical deployment necessitates a robust and instantaneous fallback mechanism to mitigate the severe QoS degradation caused by inevitable prediction errors. This validates the design of our framework, which integrates predictive intelligence with the guaranteed stability of standard 5G protocols. This result is of significant importance. It does not invalidate our predictive model but rather powerfully demonstrates the simulation-to-reality gap and underscores the absolute necessity of the robust fallback mechanism. The observed unpredictability is precisely the scenario that a real-world system must handle. It proves that for any practical deployment, predictive intelligence must be tightly coupled with the guaranteed stability of standard reactive protocols. This ensures service continuity and prevents performance degradation, especially during the initial deployment and continual learning phases of a model’s lifecycle.

#### 6.2.4 Outage probability.

**Fig 44:**
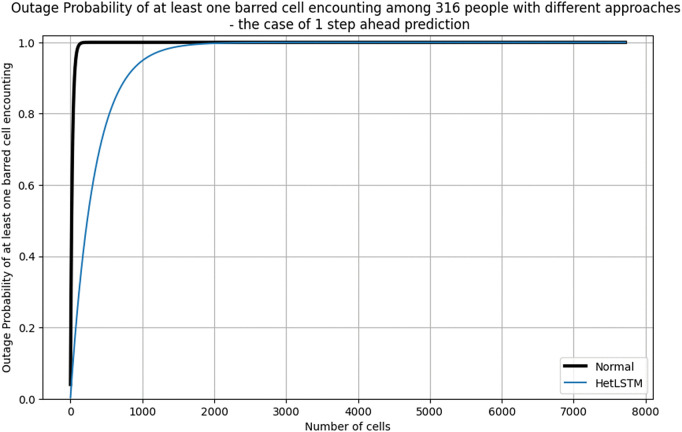
The network outage probability in the experiment case of 1-step-ahead-prediction.

**Fig 45:**
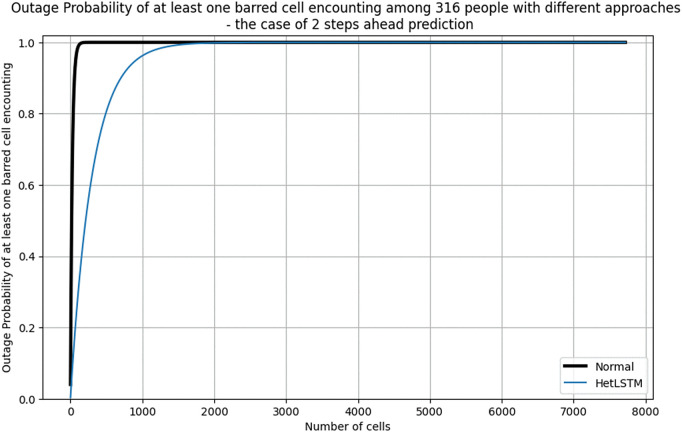
The network outage probability in the experiment case of 2-step-ahead prediction.

**Fig 46:**
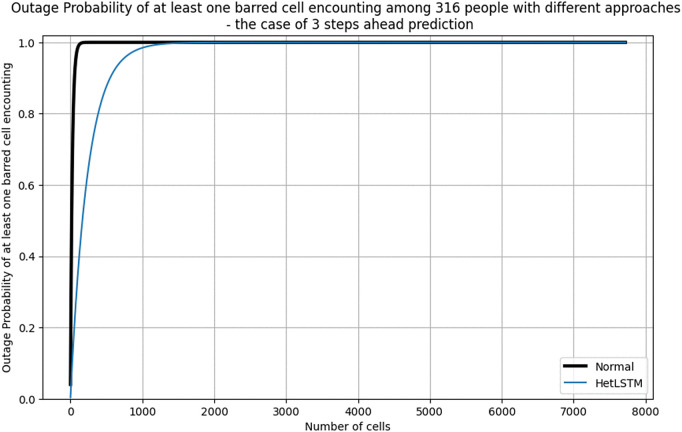
The network outage probability in the experiment case of 3-step-ahead prediction.

**Fig 47:**
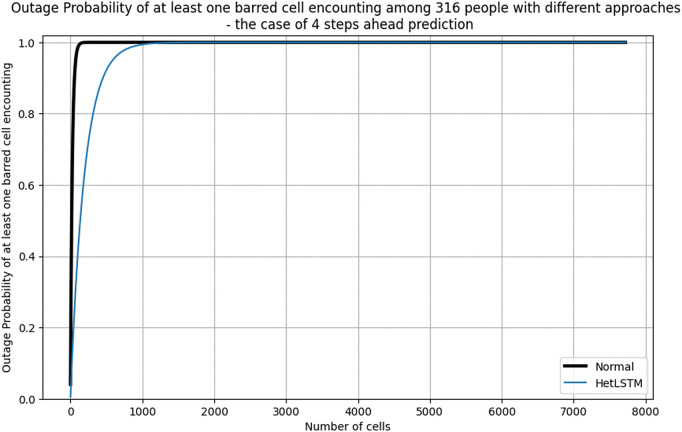
The network outage probability in the experiment case of 4-step-ahead prediction.

Another considered metric is the system’s outage probability. Figures from Figs 44–47 compare the network’s outage probability—defined as the probability that at least one user encounters a barred cell—between the Traditional (Normal) approach (black curve) and the predictive AR-LSTM approach (blue curve) across prediction horizons of 1–4 steps. From the results, we can see that:

a) *The Fragility of Reactive Networks (The Black Curve):* Across all four figures, the Normal approach curve exhibits a near-vertical rise, reaching a probability of 1.0 (certainty) with a very small number of barred cells. This indicates that in a dense, real-world network with 316 active users, the statistical likelihood of someone stumbling into a problem cell is extremely high. Without proactive guidance, the network is fragile; a barred cell is almost immediately encountered by a user, leading to service failure.
b) *The Resilience of Predictive Networks (The Blue Curve):* In stark contrast, the proposed algorithm (which is shortly labeled as HetLSTM) curve rises much more gradually. A significantly larger number of cells must be barred before the outage probability approaches 1.0. This resilience gap between the blue and black lines represents the system’s ability to successfully steer users away from barred cells. The model effectively identifies no-go zones and reroutes traffic.
c) I*nterpretation:* This suggests that while fine-grained optimization (picking the absolute fastest cell) becomes harder over time, the coarse-grained safety task (avoiding a barred cell) remains robust. The model successfully learns well enough to prevent outages, even if the precise timing of the handover creates some latency variance.

This outcome is critical to the validation of our framework. It demonstrates that despite the performance volatility observed in latency and throughput (the “cost” of prediction errors), the primary safety goal is achieved. The predictive model successfully mitigates the overall risk of network outage, proving that even a partially trained model provides a substantial reliability upgrade over standard reactive methods.

## 7 Discussion and future work

### 7.1 Purpose and operational role

**Fig 48:**
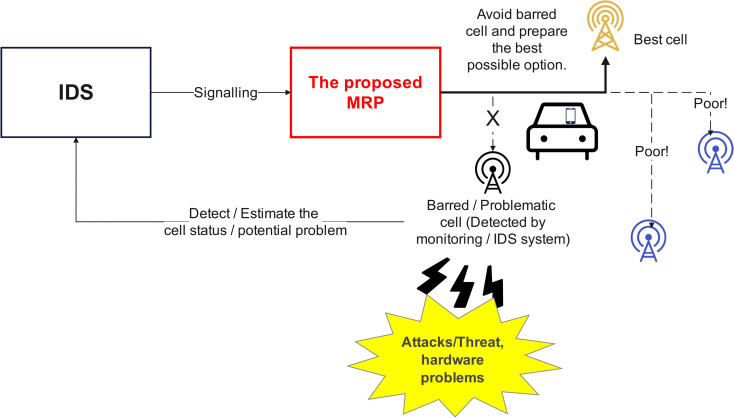
The integration and cooperation between detection – IDS and planning - Mobility and Resource prediction algorithm (MRP).

All the above is the entire proposed idea and model we suggest. Now, the purpose of the entire algorithm is to assist the system in deciding what to do when a problem is detected, or potentially happens, which contributes to create a more self-aware and automatic 5G system, oriented toward 6G in the future. The concept of “assisting the system” is fundamentally based on deciding upon a replacement cell when the monitoring system flags a potential issue. In modern 5G networks, the task of detecting anomalies and monitoring system health is typically performed by an Intrusion Detection System (IDS) [59,60]. Within the 5G Service-Based Architecture (SBA) [61] and edge computing nodes [62], the IDS is responsible for identifying threats, including abrupt malfunctions caused by electromagnetic attacks (jamming) or hardware failures [63,64]. However, what should the system do after being warned of a potential threat/problem that is highly likely to occur when the user reaches that cell position? This is where the decision suggesting plays its crucial role, to calculate and plan across all possible options, which is in the case of our proposed algorithm, estimating good, functional surrounding cells and plan for their connection based on mobility prediction (For predicting the user’s position) and resource prediction (for estimating if the cells can afford the users’ needs), in other words, planning and preparing, to tell the system what it should do to maintain the connection, avoiding where the threat/problem will happen and reduce the handover duration. Fig 48 illustrates the idea of a support system using our proposed algorithm that works after the IDS, to serve the dual role of: Detecting and Planning, to overcome a highly potential threat/problem that will happen at the cells before the users arrive. However, the framework is also designed to be robust in scenarios where the IDS provides no warning, such as during an abrupt attack. In such events, the system relies on a fundamental fallback mechanism (as discussed previously in the Users’ average latency subsection). So, in overall, if implemented, the possible operational workflow of our proposed algorithm, cooperating with the monitoring system, can be:

- The **IDS** acts as the *supervisor* of the network, detecting and warning the system about potential disruptions likely to occur in the near future.
- The **MRP algorithm** acts as the *advisor*, translating the IDS warning into actionable steps—specifically, selecting the best alternative cells and preparing the connection beforehand.

Furthermore, the preparation of the preparable connection process is a secondary benefit from the MRP algorithm, since it helps the system to plan beforehand all the preparable procedures to cut short the amount of connection establishment, contributes to a smoother transition of the handover process, and increases the QoE. With this preparation, the system can estimate the best cells with the best number of accessing users, that have enough resources to serve them, and plan to reroute the transmission more effectively than just waiting for the user to come and their UE scan for the better cells, and establish the connection step by step.

The above Planning and Preparing are what make the purpose of the Mobility and Resource prediction algorithm, and the benefit the network system gets from it is having improved the proactivity and reducing latency of the network system, which is the key to developing a self-aware, automatic, and cognitive mobile communication network in the future.

### 7.2 Encoding comparison

As mentioned above, we have used both binary encodings in our two experiments on the mobility problem. The choice of encoding scheme for categorical identifiers (User IDs and Cell IDs) is a critical design decision that directly impacts the scalability and feasibility of the entire system. We performed a quantitative comparison between the two primary candidates: one-hot encoding and binary encoding. The most significant difference lies in memory consumption. One-hot encoding generates a sparse vector of size *N* for *N* unique categories, while binary encoding creates a dense vector of size *ceil*(*log*_2_(*N*)). This difference is exponential. This theoretical advantage was confirmed in our HetNet experiment using the CRAWDAD dataset, which contained over 7,000 unique cell IDs.

**Fig 49:**
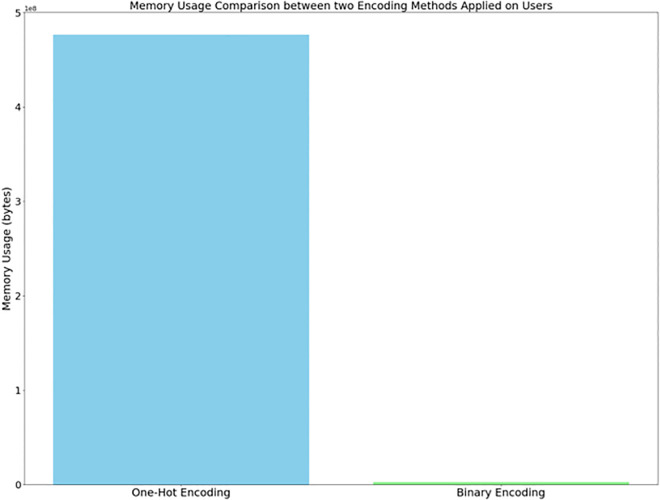
The memory usage comparison between two encoding approaches applied to users.

**Fig 50:**
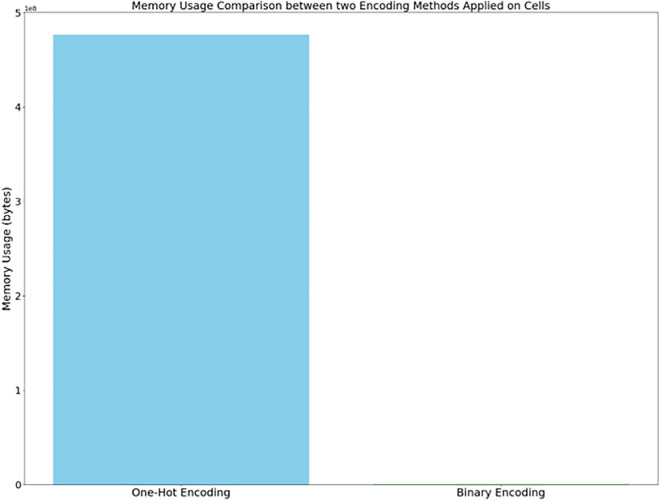
The memory usage comparison between two encoding approaches applied to cells.

**Fig 51:**
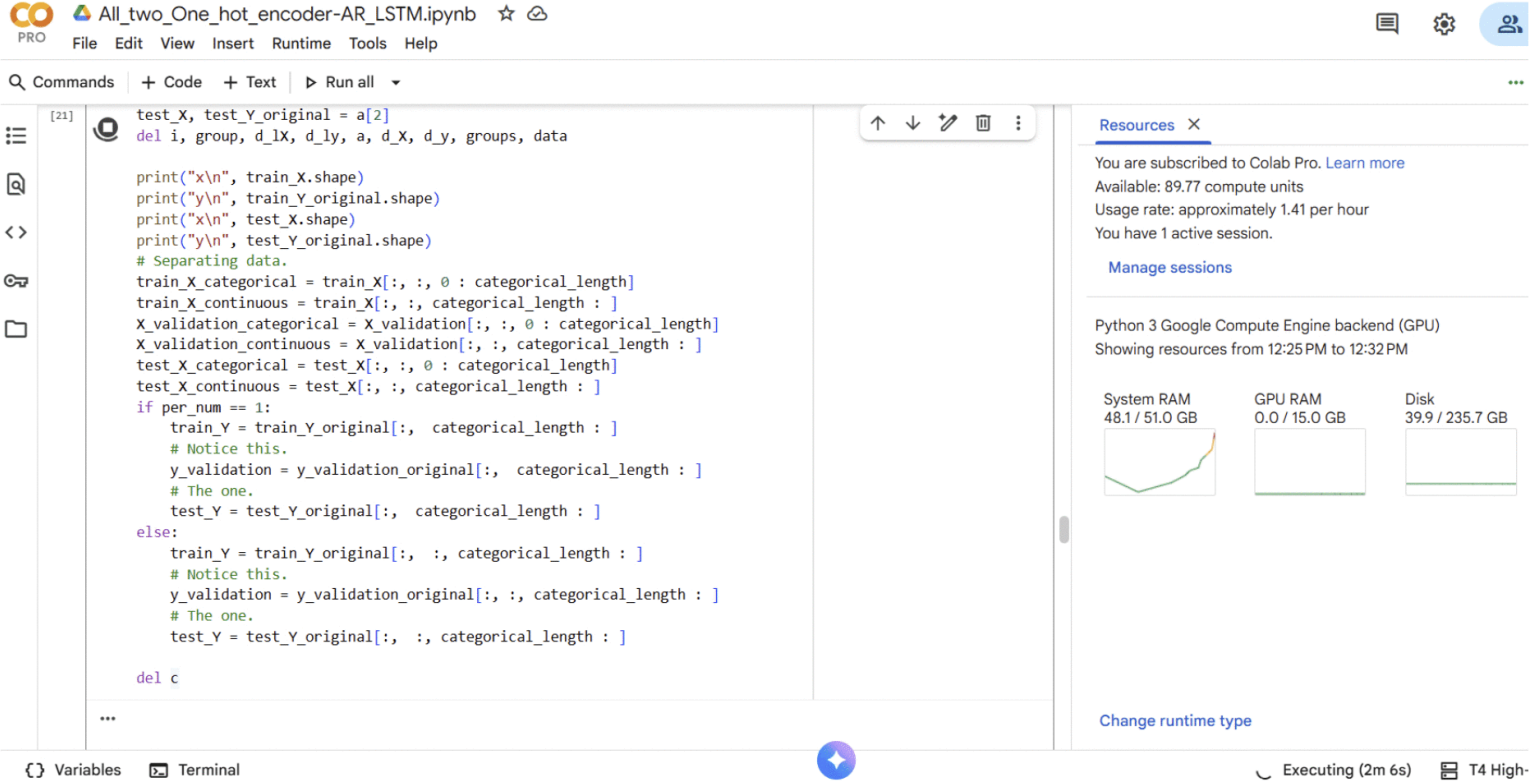
Demonstration of one-hot encoder heavy memory consumption.

**Fig 52:**
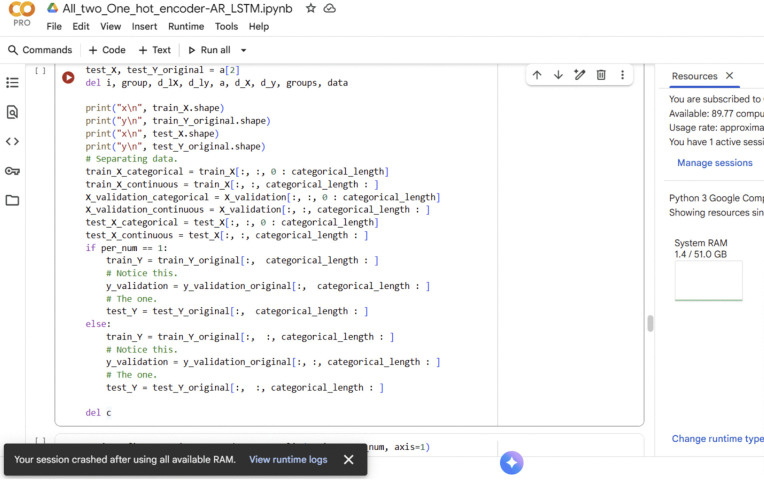
Demonstration of crash due to memory overload.

As shown in Figs 49 and 50, attempting to use one-hot encoding resulted in massive pre-processing data structures. While the base memory footprint for a subset of the one-hot-encoded variables quickly approached 500 MB ($5\times 10^{8}$ bytes), scaling this sparse matrix to the dataset’s full sequence length required more than 50 GB of system RAM. This exceeded the capacity of our experimental systems, causing out-of-memory crashes as shown in Figs 51 and 52. Consequently, one-hot encoding cannot be successfully trained at scale and is completely impractical for real-world deployment on edge devices.

In contrast, binary encoding required a vector of only 13 features (*ceil*(*log*_2_(7000)) = 13) for encoding the cells’ IDs and 9 features (*ceil*(*log*_2_(316)) = 9) for the users. As illustrated in the comparative charts in Figs 49 and 50, binary encoding reduced memory consumption by about 99% for both User and Cell IDs, making the problem computationally tractable.

This analysis of memory consumption presents a clear trade-off. While one-hot encoding might offer a slight theoretical edge in representational independence, it is computationally and memory-infeasible for any large-scale, real-world scenario like the one we are addressing. A marginal potential loss in accuracy with binary encoding is an acceptable and necessary trade-off when the alternative is a system that cannot be run at all. Therefore, we selected binary encoding as the only viable and scalable method that balances representational capacity with the practical hardware constraints of a dense mobile network.

### 7.3 The computational complexity and energy efficiency discussion

A pragmatic evaluation requires an analysis of the system’s computational overhead and energy efficiency, particularly for large-scale deployments. We analyze the complexity in two distinct phases: offline training and real-time inference.

Offline Training: The initial training of the AR-RNN model is computationally intensive, requiring large datasets and GPU-accelerated hardware. However, this is just a one-time or periodic offline cost performed on centralized servers and does not impact the real-time operational performance of the network.

Real-Time Inference: Real-time running can absolutely proceed on the CPU alone. The critical factor for deployment is the inference overhead—the cost of making a prediction for an active user at the network edge (for example, by the gNB). Because the real-time inference relies on fast matrix multiplication operations, the theoretical computational overhead of the proposed framework is exceptionally low. To rigorously quantify this, we profiled both the mobility and resource prediction models using the TensorFlow Profiler. As detailed in Table 5, a single inference step requires approximately 21,000 to nearly 29,500 Floating Point Operations (FLOPs), which is 25,250 FLOPs ≈25,000 FLOPs (rounded) on average, scaling only marginally as the prediction horizon increases from 1 to 4 steps ahead. Notably, the computational cost remains highly stable across both idealized homogeneous environments and complex, irregular HetNet deployments.

**Table 5:** Computational cost (FLOPs) of predictive models across network scenarios and prediction horizons.

Network Scenario	Steps Ahead	Mobility Prediction (Avg. FLOPs)	Resource Prediction (Avg. FLOPs)
Homogeneous (10m - 50m avg.)	1	22,504	21,575
	2	24,745	22,576
	3	28,829	23,577
	4	29,227	24,578
Heterogeneous (HetNet)	1	22,349	22,349
	2	24,364	22,576
	3	26,379	26,379
	4	28,394	24,578

To effectively handle diverse user cohorts, in the case of the HetNet experiment, our proposed algorithm utilizes a partitioned ensemble approach of approximately 22 concurrent models for 316 users (11 models for mobility prediction and 11 models for resource prediction across 316 users). The prediction for one user is computationally independent of the total number of users, meaning the computational load scales linearly. Even at peak load—evaluating hundreds of users, the total computational requirement across the ensemble remains strictly in the low MegaFLOPs range, for example, 316 users×2 predictions per user×~25k FLOPs≈15.8 MegaFLOPs, distributed across the 22 loaded models.

Energy Efficiency: Energy efficiency is critical for low-power small cell devices. In modern 3GPP 5G NR and O-RAN architectures, predictive AI/ML models operate as “xApps” within the Near-Real-Time RAN Intelligent Controller (Near-RT RIC) or the Central Unit (CU) [65,66]. Given that modern 5G edge processors routinely handle GigaFLOPs to TeraFLOPs of throughput to manage baseband processing and edge intelligence [67], an algorithmic overhead of ~15.8 MegaFLOPs utilizes less than 0.1% of a standard edge server’s real-time processing capacity. While energy consumption is strongly correlated with computational load, this mathematically negligible footprint serves as a proxy metric. Our conclusion that the framework can be safely integrated into energy-conscious femtocell deployments without compromising their power budget remains a theoretical expectation based on algorithmic size. Direct physical measurement of the electrical wattage drawn on specific 5G hardware components is outside the scope of this study and remains an important area for future experimental validation.

### 7.4 Addressing multi-UE contention

Our current simulation models users independently to isolate the prediction algorithm’s performance. However, in a dense, live network, multiple UEs often contend for limited resources in the same target cell. In such scenarios, our predictive framework serves as a critical input to the gNB’s resource scheduler. By providing advance warning of incoming resource demands from multiple users, the system transforms a reactive contention problem into a proactive one. This foreknowledge allows the scheduler to make intelligent decisions before the contention occurs, such as performing early admission control, preemptively adjusting Quality of Service (QoS) parameters for contending users, or triggering a search for alternative, less-congested target cells.

### 7.5 Continual learning and model adaptation

A critical requirement for real-world deployment is the predictive model’s ability to adapt to non-stationary environments, such as the introduction of new users or long-term shifts in urban traffic patterns. A static model trained once may degrade in performance over time. To address this, our framework leverages the inherent flexibility of the underlying neural network architecture (implemented via TensorFlow/Keras) to support continual learning.

The adaptation strategy follows an incremental learning paradigm. Instead of retraining the model from scratch—which is computationally expensive—the system periodically performs fine-tuning on the deployed model. This process involves:

Weight Retention: The pre-trained weights of the AR-RNN, which capture the fundamental mobility physics and features, are loaded as the initialization state for the new training cycle.
Incremental Updates: As new trajectory data is collected from the network, it is fed into the model using standard training routines (for example, model.load_model() and model.fit() in the Tensorflow, Keras libraries). This allows the model to adjust its weights to accommodate new spatial patterns or user behaviors.

However, a known risk in this process is catastrophic forgetting, where the model overwrites previously learned knowledge to fit the new data. To mitigate this and ensure long-term operational stability, we propose employing an Experience Replay (or Rehearsal) strategy. In this approach, the new training dataset is augmented with a small, representative subset of historical data. Additionally, a significantly lower learning rate is applied during the fine-tuning phase compared to the initial training. This ensures that the model gently adapts to new trends without destabilizing its core predictive capabilities.

To sum up, while this study establishes the potential of the AR-RNN framework, several avenues remain for future research. Our primary focus will be on expanding the findings presented in the Validation in HetNet and Real-World Mobility Data section into a full-scale performance evaluation using the heterogeneous CRAWDAD dataset, incorporating complex inter-cell interference modeling. Additionally, future work will include rigorous empirical measurements of energy consumption on physical 5G testbed hardware and the implementation of the online continual learning pipeline described above.

## 8 Conclusion

This study introduced a unified predictive mobility and resource management framework for 5G NR small-cell homogeneous networks and HetNets. By employing a highly optimized, binary-encoded AR-RNN architecture, the proposed method simultaneously predicts user trajectories and required bandwidth allocations, achieving prediction accuracies of up to 95.8%, higher than other types of mobility prediction algorithms. Then, rather than relying on reactive handover procedures, we integrate these predictions with standard network monitoring systems. This integration forms a proactive framework in cooperation with the modern monitoring systems that enables the network to preemptively perform handover preparations, mitigate transmission problems, and seamlessly route UEs around cells that are flagged for potential barring by the monitoring systems.

Our comprehensive simulations, supported by rigorous non-parametric statistical testing, demonstrate that this proactive approach significantly reduces handover latency and network outage probabilities compared to traditional reactive methods across both homogeneous and HetNet environments. Furthermore, computational profiling confirms that the AR-RNN model’s memory footprint and MegaFLOP overhead are mathematically negligible. This makes the algorithm highly feasible for real-world deployment on energy-constrained 5G edge processors without compromising their power budget. Ultimately, this framework provides a concrete, scalable, and computationally efficient solution to ensure seamless service continuity and enhanced quality of service in the development of 5G and next-generation cellular networks.
